# Excitatory neurons and astrocytes‐specific dysregulation and aberrant interactions are vulnerable to FCDI as suggested by single‐cell spatial transcriptomics

**DOI:** 10.1002/ctm2.70673

**Published:** 2026-05-01

**Authors:** Yaqian Zhang, Qihang Zou, Yingying Liu, Yinchao Li, Yubao Fang, Tiancai Huang, Jiabin Yu, Lisen Sui, Dezhi Cao, Liemin Zhou

**Affiliations:** ^1^ Department of Neurology The Seventh Affiliated Hospital Sun Yat‐Sen University Shenzhen China; ^2^ Department of Neurology Henan Provincial People' s Hospital；Zhengzhou University People' s Hospital Zhengzhou China; ^3^ Department of Neurology Third Affiliated Hospital Sun Yat‐Sen University Guangzhou China; ^4^ Department of Epilepsy Center The Second Affiliated Hospital Guangzhou University of Chinese Medicine Guangzhou China; ^5^ Department of Neurology Epilepsy Center Shenzhen Children's Hospital Shenzhen China

**Keywords:** astrocytes, excitatory neurons, focal cortical dysplasia type I, single‐nucleus RNA sequencing, spatial transcriptomics sequencing

## Abstract

**Background:**

Focal cortical dysplasia (FCD) is a common neurodevelopmental disorder characterised by cortical malformations and is a major cause of drug‐resistant epilepsy. FCD type I (FCDI) presents with architectural abnormalities of the neocortex but without cytological abnormalities. Currently, FCDI remains a significant clinical challenge.

**Methods:**

Epileptogenic cortical tissues from three FCDI patients and three relatively normal neocortical tissues as controls were analysed using single‐nucleus RNA sequencing and spatial transcriptomic for multi‐omics integration.

**Results:**

This study constructed a single‐cell spatial transcriptomic atlas of the epileptogenic cortex from FCDI patients. Excitatory neurons (ENs) and astrocytes (Ast) exhibited the most prominent alterations in FCDI. Hub genes associated with FCDI were identified in ENs, and a transcription factor (TF)‒hub gene regulatory network was constructed. Notably, *CBLN2* ^high^Ex‐1 was identified as being potentially involved in processes related to neuronal hyperexcitability and cortical development in FCDI. Western blot and immunofluorescence assays validated the altered expression of selected key genes and TFs at the protein level. Additionally, Ast exhibited increased heterogeneity, impaired differentiation and a higher proportion of immature Ast in FCDI, with predicted TFs regulating this process. Further analysis revealed aberrant signalling pathways and ligand‒receptor interactions between ENs and Ast in FCDI, with spatial co‐localisation patterns that may contribute to disease progression.

**Conclusions:**

This study highlights the specific dysregulation of ENs and Ast, along with aberrant cellular communication, which may play a critical role in the pathogenesis of FCDI. These findings provide novel insights into the molecular mechanisms underlying FCDI and offer potential therapeutic targets for precision treatment and drug development.

## Graphical abstract

• Excitatory neurons and astrocytes show marked alterations in the single‐cell spatial transcriptomic atlas of epileptogenic cortex from patients with focal cortical dysplasia type I (FCDI).

• The *CBLN2* ^high^ excitatory neuron subtype may be associated with neuronal hyperexcitability and cortical development in FCDI.

• An increased presence of immature astrocytes and their abnormal interactions with excitatory neurons in FCDI may promote disease progression.

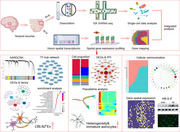

## 1 BACKGROUND

Focal cortical dysplasia (FCD) is a subtype of malformations of cortical development (MCD) and a major cause of drug‐resistant epilepsy.^1^ It is the most commonly observed pathological finding in resected brain tissue from children and young adults.^2^, ^3^, ^4^ In 2011, the International League Against Epilepsy (ILAE) introduced a stratified classification of FCD based on clinical, neuroimaging, and histopathological features.^5^ FCD type I (FCDI) is characterised by abnormal neocortical architecture, and is classified into FCDIa, FCDIb and FCDIc. In particular, FCDIa exhibits radial micro‐columnar cortical architecture,^6^ whereas FCDIb shows tangential cortical layering abnormalities, and FCDIc displays features of both. However, due to the lack of consistent neurophysiological, imaging and genetic biomarkers, the subclassification of FCDI remains controversial in both clinical and research contexts. Although the ILAE proposed a multilayered genotype‒phenotype diagnostic framework in 2022,^7^ studies on FCDI remain limited, with only few new insights updated.

FCDI is clinically characterised by focal epilepsy with onset in infancy or early childhood,^4^ frequently accompanied by developmental delay, maladaptive behaviours and neuropsychiatric comorbidities.^8^, ^9^ It typically involves multiple lobes and exhibits early resistance to antiepileptic drugs.^10^ Due to its subtle cortical structural abnormalities without significant changes in cell density, FCDI is often undetectable on neuroimaging,^11^, ^12^ posing a major diagnostic challenge. Unlike FCDII, which is characterised by genetic mutations in the mTOR pathway,^13^, ^14^, ^15^ no specific genetic alterations have been identified in FCDI, and no targeted therapeutic agents are available. Surgical resection remains the most effective treatment for drug‐resistant epilepsy.^16^ However, given the more diffuse structural abnormalities and poorly delineated epileptogenic zones in FCDI, complete lesion removal is often unachievable, leading to suboptimal postoperative outcomes.^4^, ^8^ Additionally, the epileptogenic focus may overlap with or be adjacent to eloquent cortical areas, increasing the risk of postoperative deficits in motor, visual, language and cognitive functions, making surgical treatment of FCDI epilepsy particularly challenging.^17^ Even after surgery, many patients continue to experience seizure recurrence and functional impairments. Therefore, further investigation into the pathophysiological mechanisms of FCDI is crucial for improving treatment strategies and long‐term outcomes.

In recent years, advances in multi‐omics technologies have enabled the construction of comprehensive molecular atlases of the human and mouse brain at single‐cell resolution.^18^, ^19^, ^20^, ^21^, ^22^, ^23^ Leveraging single‐cell multi‐omics approaches, these studies have provided critical insights into the molecular and cellular mechanisms of disease‐associated cell subpopulations.^24^, ^25^ Increasing evidence suggests that specific neuronal and glial subtypes play pivotal roles in the initiation and progression of epilepsy.^26^, ^27^, ^28^ However, single‐nucleus RNA sequencing (snRNA‐seq) requires tissue dissociation for cell isolation, resulting in the loss of spatial information. The integration of single‐cell sequencing with spatial transcriptomics offers a powerful strategy for disease research by simultaneously capturing cell‐type specificity and spatial localisation. This integrated approach enables the identification of cellular functions and spatial interactions within diseased tissue, offering valuable insights for the precise diagnosis and targeted treatment of neurological disorders.^29^

The roles of cortical cell types and their molecular landscape alterations in FCDI remain poorly understood. In this study, we performed snRNA‐seq and spatial transcriptomics sequencing (ST‐seq) on human brain tissue from the same cortical region to comprehensively profile cell‐type composition and disease‐associated molecular changes, along with their spatial distribution in the epileptogenic cortex of FCDI. We specifically investigated the contributions of excitatory neurons (ENs) and astrocytes (Ast) to disease pathogenesis. This integrative multi‐omics analysis provides new insights into the complex molecular networks and intercellular interactions underlying FCDI.

## 2 MATERIALS AND METHODS

### 2.1 Collection of human brain tissue samples

This study included three FCDI patients with highly epileptogenic temporal neocortex as the experimental group, two patients with benign temporal lobe tumours exhibiting non‐epileptogenic temporal neocortex adjacent to the tumour, and one patient with isolated hippocampal sclerosis and non‐epileptogenic temporal neocortex without pathological abnormalities as the control group. All participants underwent a standardised preoperative evaluation, which included detailed medical history collection, physical examination, video electroencephalography monitoring, neuropsychological testing, high‐resolution magnetic resonance imaging (3.0T) and 18F‐fluorodeoxyglucose positron emission tomography (PET‐CT) scanning. Multimodal imaging fusion was used to precisely localise the epileptogenic focus. Detailed clinical characteristics of the patients are provided in Supporting Information S1.

The identification of highly epileptogenic temporal neocortex was confirmed via preoperative stereo‐electroencephalography (SEEG, Figure S1A) and intraoperative electrocorticography (ECoG, Figure S1B). In the control group, SEEG and ECoG recordings showed no detectable epileptiform discharges. The pathological diagnoses for all patients were verified by postoperative histological examination (Figure S2). Neuropathological assessments of resected brain tissue included haematoxylin and eosin (H&E) staining, as well as immunohistochemical analyses using established markers (NeuN, GFAP, SIM‐32, CD34, NF and vimentin).^1^, ^3^ Diagnoses were independently reviewed by two senior neuropathologists, and only cases with high inter‐rater concordance were included in the study. The FCDI diagnoses adhered to the 2022 ILAE diagnostic criteria.^7^ No abnormal histopathological findings were observed in the temporal neocortex of the control group.

### 2.2 Single‐nucleus dissociation of brain tissue

Single‐nucleus dissociation was performed following the 10× Genomics protocol with optimisations based on laboratory‐specific conditions. RNA integrity of each sample was first assessed using an Agilent 2100 Bioanalyser, and only samples meeting quality criteria were processed further. Brain tissue was sectioned into 1‒2 mm^3^ pieces and transferred to a Dounce homogeniser containing lysis buffer (10 mM Tris‒HCl, pH 7.4; 10 mM NaCl; 3 mM MgCl_2_;  .1% Tween‐20;  .1% IGEPAL; 2% bovine serum albumin (BSA)). The tissue was homogenised to ensure complete lysis and nuclear release. The homogenate was transferred to a centrifuge tube and spun at 4°C for 10 min. The supernatant was removed, and the nuclear pellet was resuspended in ice‐cold wash buffer (1× phosphate‐buffered saline (PBS) with 1% BSA and  .2 U/µL RNase inhibitor) to maintain nuclear integrity. A 40 µm cell strainer was used to remove debris and large aggregates. Nuclear concentration and integrity were assessed using a hemocytometer and trypan blue exclusion staining. Myelin debris was removed using a myelin removal reagent, followed by additional centrifugation and washing steps to obtain purified nuclei. The final nuclear concentration was adjusted to 700‒1200 nuclei/µL for downstream library preparation.

### 2.3 Single‐nucleus library preparation and sequencing

Isolated nuclei were loaded onto a 10× Genomics chromium microfluidic chip and captured using the Chromium Controller. Each nucleus was encapsulated with gel beads and reagents to form single‐nucleus droplets (gel beads‐in‐emulsions, GEMs). Within the GEMs, reverse transcription (RT) was performed to generate barcoded cDNA from nuclear mRNA. The cDNA fragments were then amplified via PCR. Library construction was carried out using the 10× Genomics Single Cell 3′ v3 reagent kit, followed by sequencing on the Illumina NextSeq X Plus platform.

### 2.4 H&E staining and brightfield imaging

Tissue sections were first assessed for quality, and only those meeting the criteria proceeded to further processing. optimal cutting temperature compound (OCT)‐embedded tissue blocks were cryosectioned at a thickness of 10 µm using a Leica CM1950 cryostat under controlled conditions (‒20°C). Sections were fixed with methanol and subjected to H&E staining to visualise tissue architecture, allowing selection of optimal sections. Tissue permeabilisation was optimised using the 10× Genomics Visium Spatial Tissue Optimisation slides and reagent kits. High‐resolution imaging was performed using a Nikon ECLIPSE Ti fluorescence microscope to determine the optimal permeabilisation time.

### 2.5 Visium spatial transcriptomics

Tissue sections were placed within the capture area of the spatial gene expression slide. After H&E staining, tissue morphology was examined under a brightfield microscope, followed by permeabilisation to release cellular mRNA. The mRNA or probes hybridised with oligonucleotides containing spatial barcodes on the spots. RT was performed within the capture area to generate barcoded cDNA, which was subsequently amplified. The cDNA was fragmented to an appropriate size (200‒300 bp) using the 10× Genomics reagent kit. High‐quality libraries were obtained through PCR amplification and assessed using an Agilent 2100 Bioanalyser. The final libraries were sequenced on the Illumina NovaSeq 6000 platform for high‐throughput analysis.

### 2.6 snRNA‐seq data quality control

Quality control of FASTQ files was performed using FastQC to remove low‐quality reads and adapter sequences (Supporting Information S2). Cleaned reads were aligned to the human GRCh38 genome using the Cell Ranger pipeline (Supporting Information S3). Gene expression was quantified per cell based on unique molecular identifier (UMI) counts, generating a cell‐by‐gene expression matrix. Further quality filtering was applied based on the following five criteria: (1) each cell had to express at least 200 genes; (2) genes were retained only if expressed in at least three cells; (3) nuclei with mitochondrial gene content exceeding 10% of total reads were removed; (4) nuclei with erythrocyte gene content exceeding 5% were excluded; and (5) doublets were removed. Following quality control, a total of 48 222 high‐quality nuclei were obtained, including 19 254 from FCDI patients and 28 968 from controls (Figure S3 and Supporting Information S4). The number of detected genes per sample ranged from 27 262 to 29 664, highlighting the high quality of our snRNA‐seq dataset. No significant batch effects were observed based on sex, age or disease duration (Figure S4). Finally, data were standardised and normalised using LogNormalise to generate a refined expression matrix for downstream analyses.

### 2.7 ST‐seq data quality control

Raw BCL files obtained from the sequencer were converted into FASTQ format using space ranger mkfastq, and low‐quality sequences were removed using FastQC (Supporting Information S5). Spatial barcode decoding was performed using Space Ranger from 10× Genomics Visium, aligning reads to the human GRCh38 genome and generating a gene expression count matrix with spatial annotations (Supporting Information S6). Spots with fewer than 100 detected genes were excluded during quality filtering. Following quality control, a total of 13 749 high‐quality spots were retained, including 5235 from FCDI patients and 8514 from controls (Figure S5 and Supporting Information S7). Data were standardised and normalised with SCTransform to generate a refined expression matrix for downstream analyses.

### 2.8 Dimensionality reduction and clustering analysis

Following standardisation and normalisation of snRNA‐seq and ST‐seq data, the top 2000 highly variable genes were selected using FindVariableFeatures. Principal component analysis (PCA) was performed with RunPCA, and the top 20 principal components (PCs) were selected based on ElbowPlot (dims = 1:20). Batch effect correction was then performed using Harmony on the first 20 PCs, using sample identity as the batch variable. A *k*‐nearest neighbour graph was constructed using FindNeighbors (dims = 1:20) in Seurat based on the Harmony‐corrected embeddings. Clustering analysis was performed using FindClusters, with the resolution parameter set to  .6. Dimensionality reduction and visualisation were conducted using RunUMAP based on the Harmony‐corrected embeddings (n.neighbors = 30, min.dist = .3). For ST‐seq data, spatial transcriptomic maps were integrated to provide spatial visualisation of clustering patterns.

### 2.9 Cell‐type annotation

Cell‐type annotation was performed by identifying differentially expressed genes (DEGs) for each cluster using FindMarkers in Seurat. Known marker genes were obtained from publicly available databases such as CellMarker and PanglaoDB to assign cell identities. Marker gene expression was visualised on uniform manifold approximation and projection (UMAP) plots to facilitate initial cell‐type identification. The expression profiles of marker genes within each cluster were analysed to refine and confirm cell‐type assignments.

### 2.10 Integration of snRNA‐seq and ST‐seq data

To integrate snRNA‐seq and ST‐seq data, gene expression matrices were aligned to ensure consistency in gene lists. Spatial deconvolution of ST‐seq data was performed using the CARD model, leveraging cell‐type signatures from snRNA‐seq data DEGs identified in snRNA‐seq were used to construct a cell‐type‐specific expression matrix. The CARD autoregressive model, combined with Bayesian estimation, was applied to infer the proportion of cell types at each spatial location in ST‐seq data. SpatialFeaturePlot was used to visualise the spatial distribution of cell types and DEGs across specific regions. Cell‒cell communication in snRNA‐seq data was analysed using CellChat and NicheNet, and interaction patterns were mapped onto spatial locations to explore region‐specific cellular interactions.

### 2.11 Gene Set Variation Analysis

Gene Set Variation Analysis is an unsupervised method for assessing the enrichment of gene sets across different samples. Using marker genes from single‐cell sequencing data to define cell types in spatial transcriptomics is an efficient approach. This analysis integrates cell‐type‐specific gene sets identified in single‐cell data with spatial gene expression profiles, enabling the identification of potential cell types across different spatial regions.

### 2.12 Gene Ontology enrichment analysis

Gene Ontology (GO) is a structured and standardised biological knowledge base designed to describe the biological characteristics and functions of genes and their products. GO analysis primarily includes three domains: cellular components, biological processes and molecular functions. GO enrichment analysis was performed using the enrichGO() function from the R package clusterProfiler. Pathways and biological functions with a significance threshold of *p* < .05 were considered enriched and were visualised based on their *p*‐values.

### 2.13 Kyoto Encyclopedia of Genes and Genomes enrichment analysis

Kyoto Encyclopedia of Genes and Genomes (KEGG) is a comprehensive database integrating genomic, chemical, metabolic and disease‐related information. KEGG enrichment analysis identifies enriched pathways by comparing detected genes to predefined pathway gene sets within the database. This analysis was conducted using the enrichKEGG() function from the R package clusterProfiler. Pathways with a significance threshold of *p *< .05 were considered enriched and were visualised based on their *p*‐values.

### 2.14 Protein‒protein interaction network analysis

Protein‒protein interaction (PPI) network analysis was performed by inputting target gene‐associated proteins into the STRING database (https://stringdb.org). DEG‐associated protein interactions were analysed using the STRING online platform. The interaction data generated by STRING were then imported into Cytoscape for further visualisation and analysis of the PPI network.

### 2.15 High‐dimensional weighted gene co‐expression network analysis

To investigate gene modules associated with FCDI, high‐dimensional weighted gene co‐expression network analysis (hdWGCNA) was applied to ENs.^30^ Gene expression normalisation was performed on the SCT matrix using the AverageExpression function in Seurat, followed by the construction of a scale‐free topology network. Low‐variability genes (variance<3) were filtered out to remove low‐expression features. A soft‐thresholding power (*β*) was selected to achieve a scale‐free topology fit of *R* ^2^ = .9. A network was constructed based on Pearson correlation, and a signed Topological Overlap Matrix was used for module detection. Modules were merged based on module eigengene (ME) correlation, with a merging threshold of  .2 and a minimum module size of 50 genes. The ‘grey’ module, representing unassigned genes, was excluded from further analysis. Correlation analysis between identified modules and FCDI phenotypes was performed, defining FCDI‐associated modules as those with a correlation coefficient (|*r*| > .05 and *p* < .05). Hub genes within each module were defined as those with a module membership score (kME) > .8. The top 10% of genes with the highest connectivity in each module were designated as hub genes, representing the core expression characteristics of the module. Cytoscape was used to construct hub gene interaction networks, illustrating intra‐ and inter‐module relationships. Finally, GO enrichment analysis was performed on hub genes within each module to identify significant biological processes and pathways associated with FCDI.

### 2.16 Transcriptional regulatory network analysis using pySCENIC

Transcriptional regulatory network (TRN) analysis in ENs and Ast was performed using the Python package pySCENIC. pySCENIC reconstructs gene regulatory networks and identifies active transcription factors (TFs). The analysis workflow consisted of the following steps: first, the core dependency package GENIE3 was used to infer potential target genes for each TF based on co‐expression relationships. Next, RcisTarget was applied to analyse co‐expression modules, identify cis‐regulatory motifs and filtering direct target genes that were significantly enriched and regulated by upstream TFs. Finally, the AUCell algorithm was used to score the expression of these genes, quantify TF activity and classify cells based on their regulatory activity. The TF‒hub gene regulatory network was visualised using Cytoscape, enabling the exploration of key regulatory interactions.

### 2.17 Pseudotime trajectory analysis

Single‐cell pseudotime trajectory analysis of Ast subpopulations was performed using the Monocle2 R package to investigate cellular dynamics during development and disease progression. First, setOrderingFilter was used to select highly variable genes, followed by dimensionality reduction using the DDRTree method to extract the continuous trajectory of cellular transitions. The orderCells function was then applied to construct the trajectory in reduced‐dimensional space and assign pseudotime values to each cell. Cell trajectories and branching structures along pseudotime were visualised using plot_cell_trajectory. BEAM analysis was performed to identify genes associated with cell differentiation at trajectory branch points, and the results were visualised in heatmaps. Finally, plot_genes_in_pseudotime was used to illustrate the expression dynamics of genes of interest along the pseudotime trajectory, generating trend curves for gene expression changes over pseudotime.

### 2.18 Cell‒cell communication analysis

Cell‒cell communication networks between ENs and Ast subpopulations in snRNA‐seq data were analysed using CellChat v2. All results were annotated based on the human ligand‒receptor database. First, identifyOverExpressedInteractions was used to filter effective ligand‒receptor pairs, while computeCommunProb and computeCommunProbPathway were applied to calculate communication probabilities between ligand‒receptor pairs and their associated signalling pathways. The aggregateNet function was then used to generate an overall communication network between cell types. Finally, cell‐type interactions were visualised using netVisual_bubble and netVisual_circle, generating bubble plots and chord diagrams illustrating intercellular signalling relationships. For ST‐seq data, the Seurat function SpatialFeaturePlotBlend was used to visualise the spatial co‐expression patterns of ligand‒receptor pairs.

### 2.19 Western blot analysis

Brain tissue samples from six patients were homogenised in RIPA lysis buffer (Beyotime) and centrifuged at 12 000 rpm for 10 min at 4°C. The supernatant was collected, and equal amounts of protein were separated by 10% sodium dodecyl sulphate‒polyacrylamide gel electrophoresis (SDS‒PAGE) and transferred onto  .22 µm polyvinylidene fluoride (PVDF) membranes. The membranes were blocked with 5% skim milk in blocking buffer (10 mM Tris, pH 7.6, 150 mM NaCl) for 1 h at room temperature. Primary antibody incubation was performed overnight at 4°C using the following antibodies: CBLN2 (1:500, SAB), NTRK2 (R&D Systems), RFX3 (Huaan Biotechnology), MEF2C (Huaan Biotechnology) and β‐actin (Three Eagles). The membranes were then incubated with horseradish peroxidase (HRP)‐conjugated goat anti‐rabbit or goat anti‐mouse secondary antibodies (1:2000, CST) for 1 h at room temperature. Protein bands were detected using an enhanced chemiluminescence substrate (Yeasen) and imaged with a Bio‐Rad gel imaging system. Band intensities of target proteins were quantified using ImageJ software for further statistical analysis.

### 2.20 Immunofluorescence

Brain tissue paraffin blocks fixed in 4% paraformaldehyde were sectioned into 5 µm slices. Sections were deparaffinised with xylene and rehydrated through a graded ethanol series. Antigen retrieval was performed by heating the sections in 1× ethylenediaminetetraacetic acid (EDTA) buffer (pH 8.0, Beyotime) using a microwave for 20 min. Endogenous peroxidase activity was quenched with 3% H_2_O_2_, and cell membrane permeabilisation was achieved using  .5% Triton X‐100 (Beyotime). Sections were then blocked with 10% goat serum at room temperature for 1 h. A mixture of two primary antibodies from different host species was applied and incubated overnight at 4°C. The primary antibodies included CBLN2 (R&D Systems), NTRK2 (R&D Systems) and NeuN (Three Eagles). For secondary antibody incubation, sections were incubated for 1 h at room temperature in the dark with Alexa Fluor 488‐conjugated anti‐mouse secondary antibody (1:100) for mouse‐derived primary antibodies and Alexa Fluor 594‐conjugated anti‐rabbit secondary antibody (1:100) for rabbit‐derived primary antibodies. Finally, sections were mounted with antifade mounting medium containing DAPI (Solarbio). Whole‐slide scanning was performed using an Olympus SLIDEVIEW VS200 scanner. Semi‐quantitative analysis of selected target regions was conducted using ImageJ software.

### 2.21 Statistical analysis

Data analysis and visualisation were performed using GraphPad Prism (version 9.0), R (version 4.3.1) and Python. Quantitative data are presented as mean ± standard deviation, while qualitative data are expressed as percentages. Normality was assessed using the Shapiro‒Wilk test, and homogeneity of variances was evaluated using Levene's test. For comparisons between two groups, an independent‐samples *t*‐test was applied when data were normally distributed. For non‐normally distributed data, the Wilcoxon rank‐sum test was employed. Comparisons of qualitative variables were performed using the chi‐square test. A two‐sided *p*‐value < .05 was considered statistically significant.

## 3 RESULTS

### 3.1 Multi‐omics analysis reveals diversity differences in human temporal neocortical cells

**FIGURE 1:**
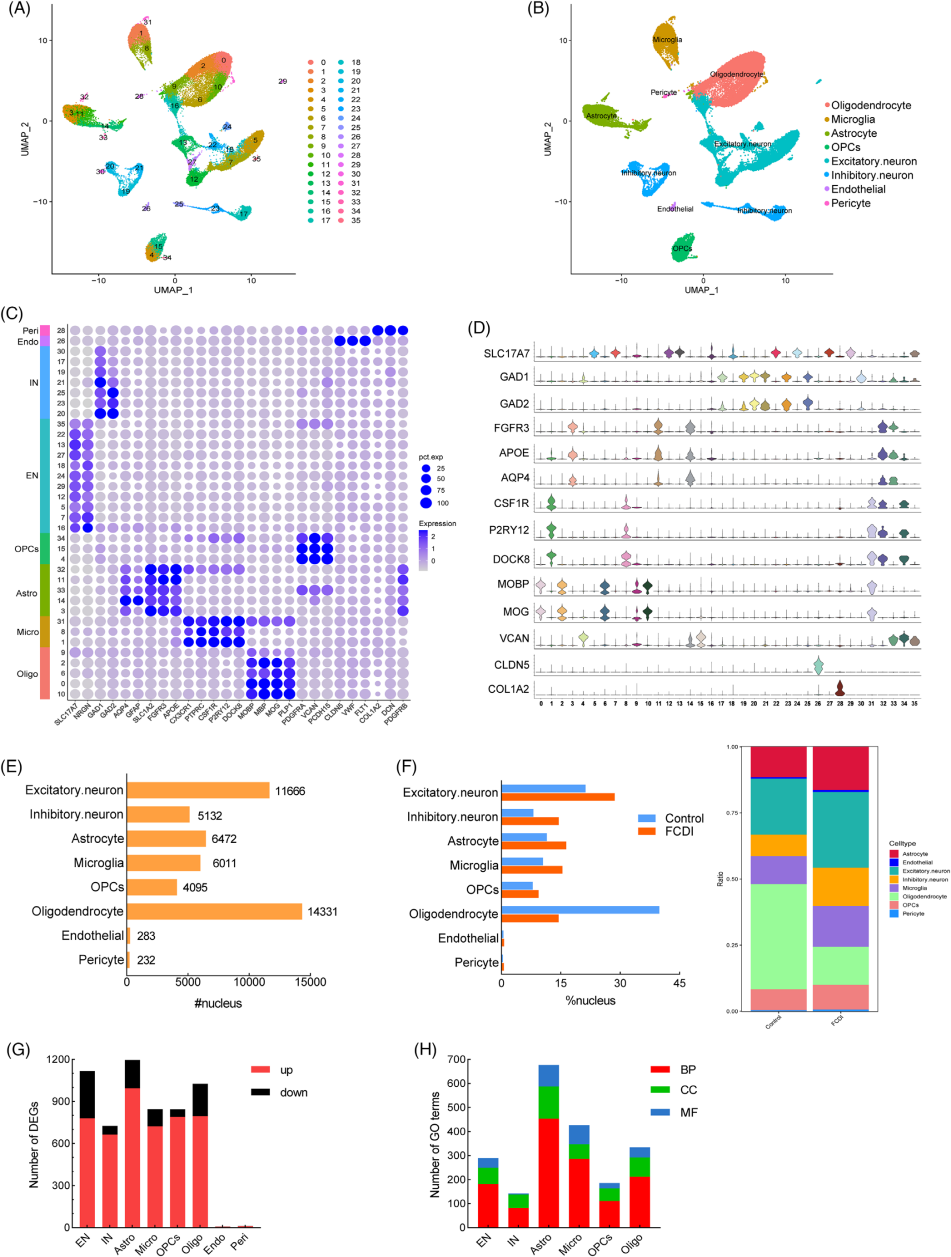
Single‐nucleus RNA sequencing (snRNA‐seq) reveals differences in cellular diversity in focal cortical dysplasia type I (FCDI) neocortex. (A) UMAP plot after dimensionality reduction clustering of the snRNA‐seq dataset, with cell nuclei coloured by cluster. (B) Cell types defined according to known marker genes, UMAP plot visualised with nuclei coloured by cell type. (C) Dot plots showing the expression patterns of known marker genes in major cortical cell types. The size of each dot corresponds to the proportion of cells expressing the gene, while the colour indicates the level of gene expression. (D) Violin plots of known marker genes expressed in different clusters. (E) Number of nuclei in the major cell types. (F) Ratio of major cell types in the FCDI and control groups. (G) Number of differentially expressed genes (DEGs) in major cell types. Wilcoxon rank‐sum test, |avg_log2FC| ≥  .1, adjusted *p*‐value < .05. (H) Gene Ontology (GO) functional enrichment of DEGs.

We performed 10× Genomics snRNA‐seq on epileptogenic temporal neocortical tissue from three FCDI patients and non‐epileptogenic temporal neocortical tissue from three control individuals. Unsupervised dimensionality reduction and clustering identified 36 distinct cell subpopulations, which were visualised using UMAP (Figure 1A). Based on characteristic marker gene expression patterns, we annotated eight major brain cell types (Figure 1B). Specifically, we identified ENs (24.19% of nuclei, SLC17A7 and NRGN), inhibitory neurons (10.64%, GAD1 and GAD2), Ast (13.42%, AQP4, GFAP, FGFR3 and APOE), microglia (12.46%, CX3CR1, PTPRC, CSF1R, P2RY12 and DOCK8), oligodendrocytes (29.71%, MOBP, MBP, MOG and PLP1), oligodendrocyte precursor cells (8.49%, PDGFRA, VCAN and PCDH15), endothelial cells (.58%, CLDN5, VMF and FLT1) and pericytes (.48%, COL1A2, DCN and PDGFRB) (Figure 1C,D). Next, we quantified the number and proportion of nuclei in each major cell type (Figure 1E,F). Compared to controls, FCDI patients exhibited a significant increase in the proportion of ENs, an increased proportion of Ast among glial cells, and a reduced proportion of oligodendrocytes. Furthermore, differential expression analysis across all cell types followed by GO enrichment analysis revealed that ENs and Ast exhibited the most pronounced transcriptomic alterations (Figure 1G,H). These findings suggest that transcriptomic abnormalities in ENs and Ast may play a crucial role in the pathological mechanisms underlying FCDI and may provide novel insights into its etiology.

**FIGURE 2:**
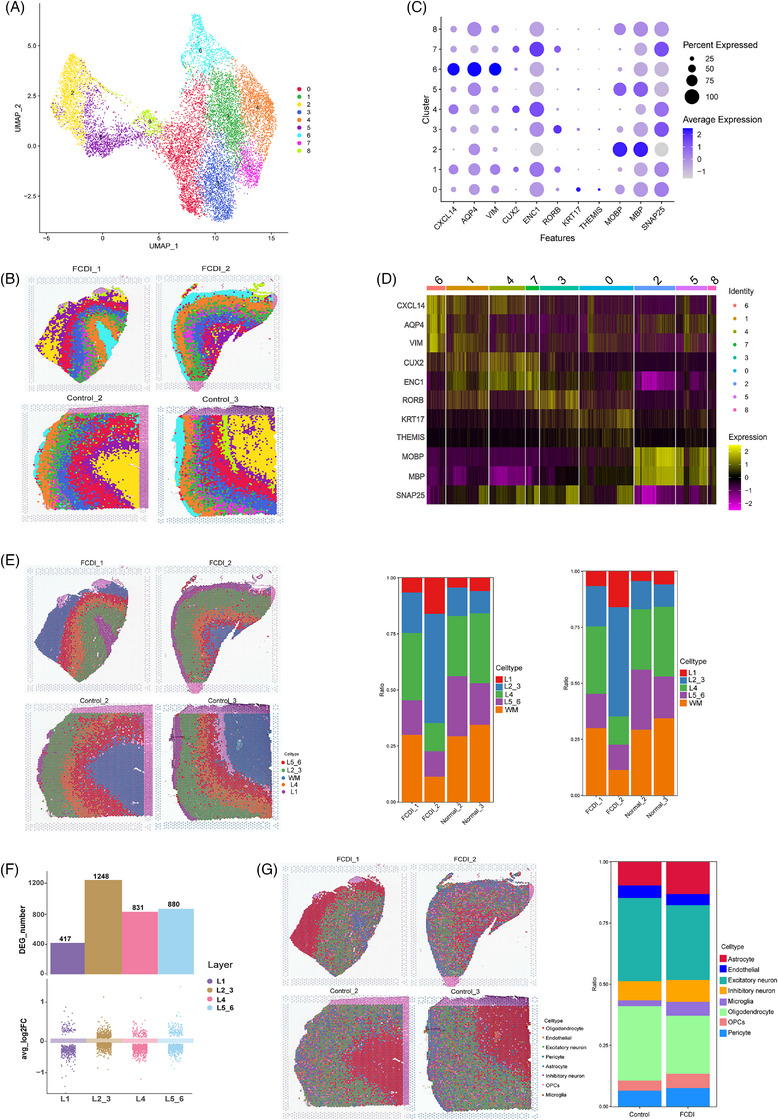
Spatial transcriptomics sequencing (ST‐seq) reveals differences in cellular diversity in focal cortical dysplasia type I (FCDI) neocortex. (A) UMAP visualisation of the ST‐seq dataset, coloured by spot assignment. (B) Spatial distribution of different clusters in the cortex. (C) Dot plots showing the expression of known marker genes in different cortical layers. The dot size corresponds to the proportion of cells expressing the gene and the colour indicates the level of expression. (D) Heatmap showing the expression of known marker genes in different cortical layers, the colour indicates the expression level. (E) Spatial distribution and proportion of different cortical regions. (F) Number of differentially expressed genes (DEGs) in different cortical layers. Wilcoxon rank‐sum test, |avg_log2FC| ≥  .1, adjusted *p*‐value < .05. (G) Spatial distribution and percentage of major cell types.

We performed 10× Genomics ST‐seq analysis on epileptogenic temporal neocortical tissue from two FCDI patients and relatively normal temporal neocortical tissue from two control individuals. After quality control, alignment, UMI filtering and unsupervised dimensionality reduction clustering, we identified nine distinct subclusters, which were visualised using UMAP (Figure 2A) and spatially mapped (Figure 2B). Based on representative cortical marker genes (Figure 2C,D), we delineated cortical regions as follows: layer I (L1) was defined by CXCL14, AQP4 and VIM; layers II‒III (L2_3) by CUX2 and ENC1; layer IV (L4) by RORB; layers V‒VI (L5_6) by KRT17 and THEMIS; and white matter (WM) by MOBP and MBP. Cluster 6 was localised to L1, clusters 1, 4 and 7 to L2_3, cluster 3 to L4, cluster 0 to L5_6, and clusters 2 and 5 to WM, resulting in eight subclusters across six cortical regions (Figure 2E). Cluster 8 exhibited variable spatial localisation with a low proportion, making its classification uncertain at this stage. Notably, FCDI patients exhibited an increased proportion of cells in cortical layers L2_3, irrespective of WM composition. Additionally, DEG analysis revealed the most pronounced transcriptional changes in L2_3 of FCDI patients compared to controls (Figure 2F), suggesting a potential pathological role for this layer in FCDI. To further deconvolute spatial transcriptomic data, we applied CARD analysis, integrating cell‐type signatures from snRNA‐seq. Using DEGs from snRNA‐seq as references, we mapped cell types to spatially captured regions, identifying eight major cell types (Figure 2G): ENs, inhibitory neurons, Ast, microglia, oligodendrocytes, oligodendrocyte precursor cells, endothelial cells and pericytes. These cell types displayed distinct spatial distributions within the cortex, generating a spatial single‐cell transcriptomic atlas. Importantly, FCDI patients exhibited significant alterations in the proportions of ENs and Ast compared to controls, underscoring their potential involvement in FCDI pathophysiology.

By integrating snRNA‐seq and ST‐seq data, we hypothesise that dysregulated ENs and Ast may contribute to the onset and progression of epilepsy in FCDI. Consequently, we focus on these cell types for further investigation to elucidate their roles in the pathogenesis of FCDI.

### 3.2 Regulatory mechanisms of EN‐specific genes

#### 3.2.1 hdWGCNA identifies gene modules associated with FCDI

**FIGURE 3:**
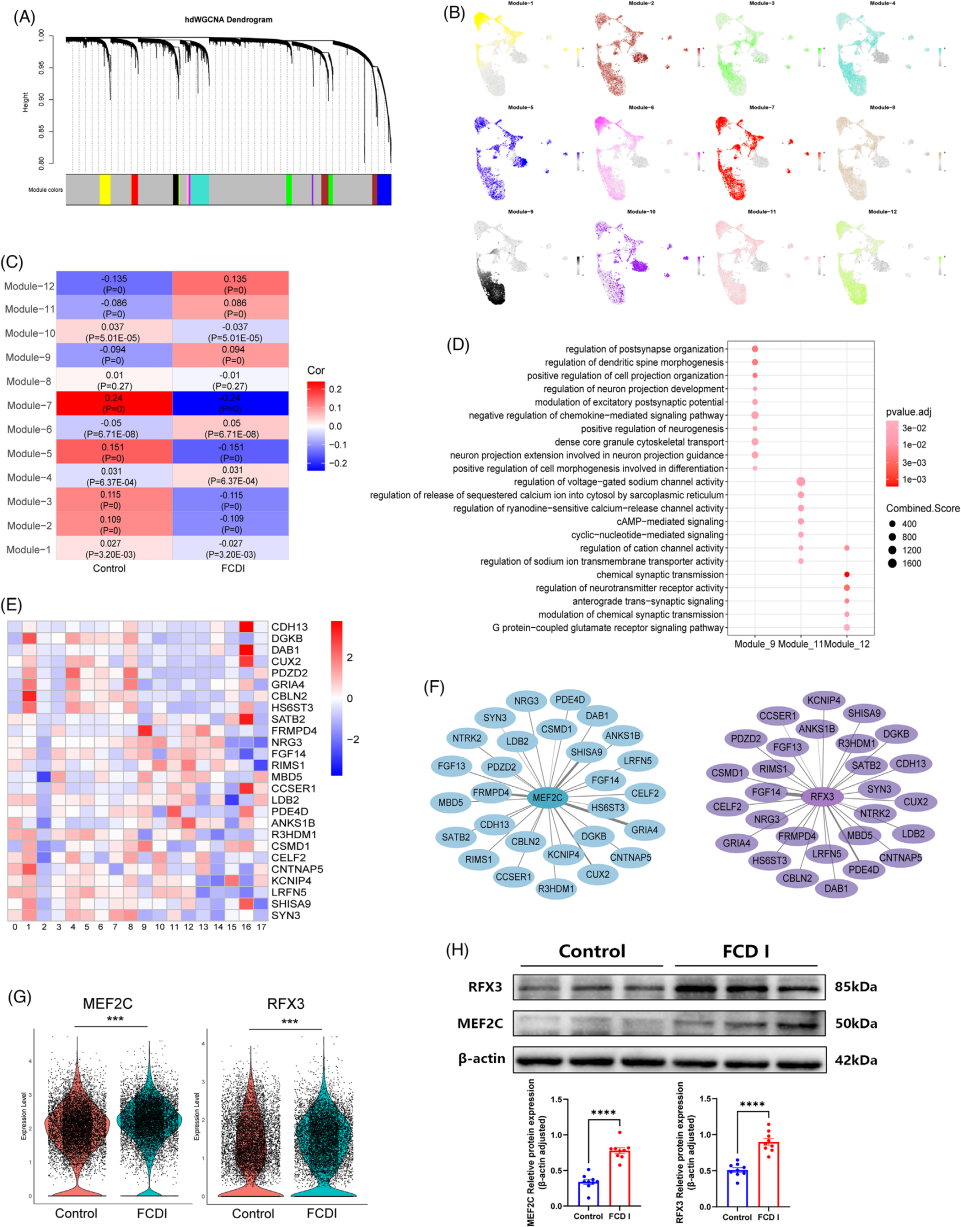
Construction of a transcription factor (TF)‒hub genes regulatory network in excitatory neurons (ENs). (A) Dendrogram of high‐dimensional Weighted Gene Co‐expression Network Analysis (hdWGCNA) module clustering. (B) Distribution of co‐expression module trait genes in UMAP. (C) Heatmap of module‐trait correlations. (D) Bubble plots of Gene Ontology (GO) functional enrichment analysis of focal cortical dysplasia type I (FCDI)‐related gene modules. (E) Heatmap of the distribution of differential expression of the hub genes of FCDI‐related modules in different clusters. Vertical coordinates represent genes and horizontal coordinates represent clusters. Colour shades represent avg_logFC, red indicates upregulation of gene expression and blue indicates downregulation of gene expression. (F) TF‒hub genes regulatory network. (G) Comparison of mRNA expression levels of the transcription factors MEF2C and RFX3 between the FCDI and control groups. Wilcoxon rank‐sum test showed adjusted *p*‐value of 1.59E‐76 for MEF2C and 5.57E‐21 for RFX3. (H) Comparison of protein expression levels of the transcription factors MEF2C and RFX3 between the FCDI and control groups. Three samples per group, and all experiments were repeated three times. An independent *t*‐test was performed, and a two‐sided *p*‐value < .05 was considered significant. MEF2C, *t* = 8.048; RFX3, *t* = 6.948. ^**^ *p* < .01,^***^ *p* < .001,^****^ *p* < .0001.

After secondary dimensionality reduction and clustering of ENs, we identified 18 subclusters (designated ‘Ex’; Figure S6A). To uncover gene modules and hub genes associated with FCDI, we performed hdWGCNA on ENs, identifying 12 distinct gene modules (Figures 3A and S6B,C and Supporting Information S8) along with co‐expression networks of hub genes within each module (Figure S7). We then mapped module‐specific genes onto the snRNA‐seq dataset and visualised their distribution via UMAP (Figure 3B). Correlation analysis between MEs and FCDI phenotypic data revealed that Module‐9, ‐11 and ‐12 exhibited significant positive correlations with the FCDI phenotype (Figure 3C and Supporting Information S9). GO enrichment analysis showed that Module‐9 genes were predominantly involved in postsynaptic structural organisation, dendritic spine morphogenesis, neuronal projection development, excitatory postsynaptic potential and cytoskeletal transport, highlighting their importance in synaptic development and plasticity. Module‐11 genes were enriched in synaptic signalling and the regulation of ion channel (Na^+^, Ca^2+^) activity, whereas Module‐12 genes were associated with chemical synaptic transmission, neurotransmitter receptor activity and G‐protein‐coupled glutamate receptor signalling. Together, these modules contain genes that play central roles in neurotransmitter release and signal transduction. Collectively, these findings suggest that key genes in Module‐9, ‐11 and ‐12 may contribute critically to the pathogenesis and progression of FCDI.

#### 3.2.2 Construction of the TF‒hub genes transcriptional regulatory network

To identify hub genes associated with FCDI, we further analysed the hub genes within Module‐9, ‐11 and ‐12. Mapping these hub genes onto EN subclusters revealed that their expression changes were most pronounced in the Ex‐1 subcluster (Figure 3E and Supporting Information S10), suggesting a potential key role of Ex‐1 in the onset and progression of FCDI. TFs are master regulators of gene expression, orchestrating complex transcriptional programs critical for neurodevelopment and neuronal homeostasis. To identify active TFs in ENs, we performed pySCENIC analysis on DEGs, followed by validation using the JASPAR database. This approach identified 230 TFs with significantly altered activity (Supporting Information S11 and S12), including key regulators of neuronal migration, differentiation, axon guidance, pruning and remodelling, such as MYT1L, CUX2, MEF2C and RFX3. To further explore the regulatory effects of TFs on hub genes in FCDI, we constructed a TRN (Figure S8). Among the candidate TFs, MEF2C and RFX3 were found to regulate multiple hub genes (Figure 3F and Supporting Information S13). Notably, both snRNA‐seq and ST‐seq datasets consistently showed elevated transcript levels of MEF2C and RFX3 in FCDI patients (Figures 3G and 5C and Supporting Information S14). Furthermore, WB analysis further confirmed significantly increased protein expression of MEF2C and RFX3 in epileptogenic neocortical tissue from FCDI patients (Figure 3H).

#### 3.2.3 *CBLN2* ^high^Ex‐1 implicates neuronal excitability and cortical development in FCDI

**FIGURE 4:**
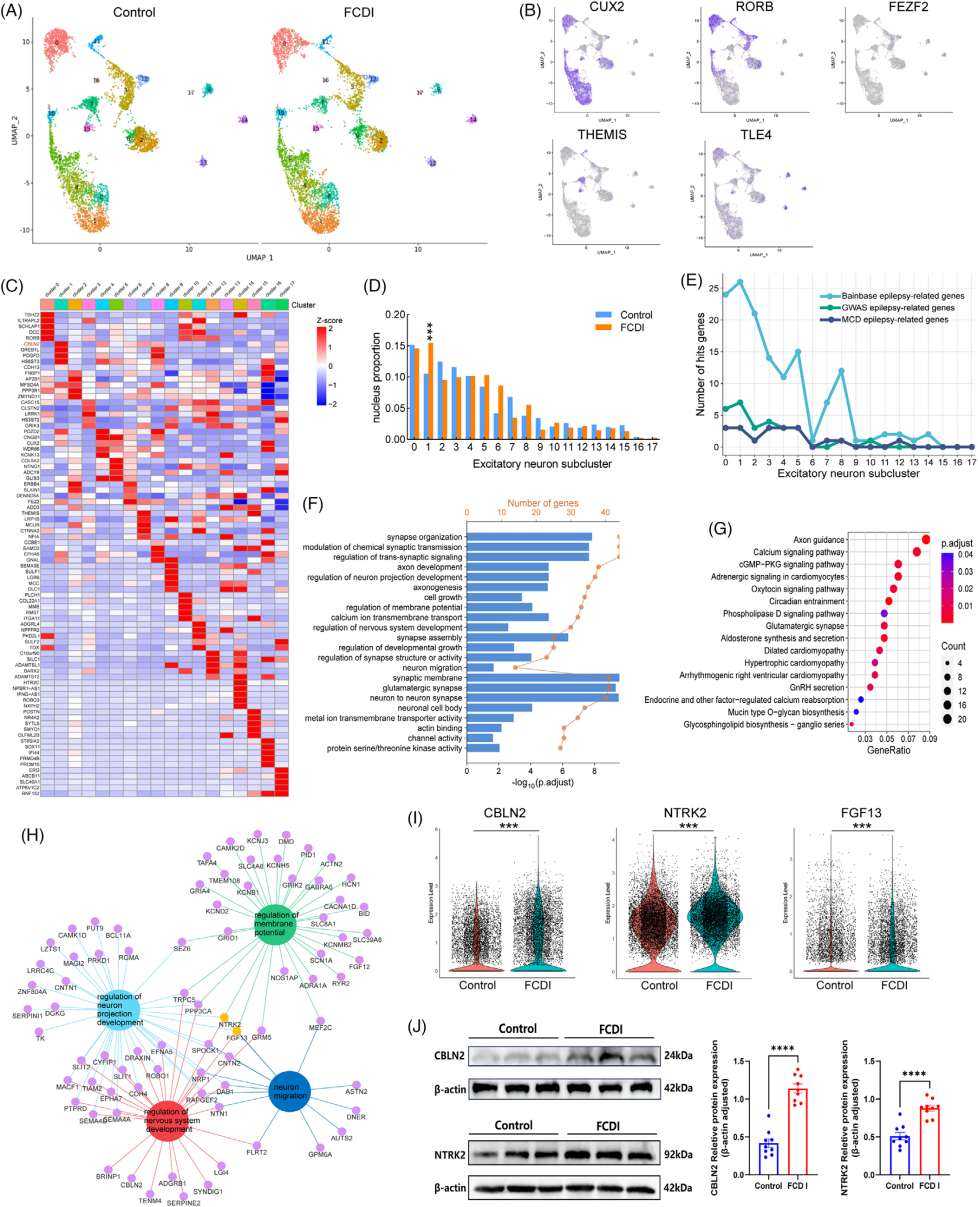
Characteristics of *CBLN2* ^high^Ex‐1 subgroup in the epileptogenic cortex of focal cortical dysplasia type I (FCDI). (A) UMAP plots of excitatory neurons after secondary dimensionality reduction clustering, with nuclei coloured by clusters. (B) UMAP plots of the distribution of known marker genes in different cortices. (C) Heatmap of top five highly expressed genes in each cluster. (D) Percentage of the number of different clusters in the FCDI and control groups. A chi‐square test was performed, and a two‐sided *p*‐value < .05 was considered significant. Ex‐1, *χ* ^2^ = 68.70, *p* < .001. (E) Line plots of epilepsy‐related gene databases mapped to differentially expressed genes (DEGs) of different clusters. (F) The Gene Ontology (GO) enrichment analysis of DEGs in the *CBLN2* ^high^Ex‐1 subgroup. (G) The Kyoto Encyclopedia of Genes and Genomes (KEGG) enrichment bubble plot of DEGs in the *CBLN2* ^high^Ex‐1 subgroup. (H) Venn diagram of the intersection of DEGs in GO‐enriched terms related to FCD. (I) Comparison of mRNA expression levels of CBLN2, NTRK2 and FGF13 between the FCDI and control groups. Wilcoxon rank‐sum test showed adjusted *p*‐value of 6.76E‐59 for CBLN2, 6.77E‐63 for NTRK2 and 2.30E‐38 for FGF13. (J) Comparison of protein expression levels of CBLN2, NTRK2 between the FCDI and control groups. Three samples per group, and all experiments were repeated three times. An independent *t*‐test was performed, and a two‐sided *p*‐value < .05 was considered significant. CBLN2, *t* = 8.819; NTRK2, *t* = 5.994. ^***^ *p* < .001,^****^ *p* < .0001.

To elucidate the role of ENs in FCDI, we conducted secondary dimensionality reduction and visualised the results using UMAP (Figure 4A). Eighteen subclusters were identified, exhibiting distributions consistent with known cortex‐related gene expression profiles (Figure 4B). For example, CUX2, a marker gene specific to cortical layers II‒III, was predominantly enriched in Ex‐1, Ex‐2, Ex‐4, Ex‐5, Ex‐6, Ex‐8 and Ex‐16. RORB, a canonical marker for layer IV, was largely expressed in Ex‐0, Ex‐3, Ex‐10, Ex‐11 and Ex‐12. Similarly, FEZF2, THEMIS and TLE4, known markers for layers V‒VI, were highly expressed in Ex‐7, Ex‐9, Ex‐13, Ex‐14 and Ex‐17. DEGs were identified for each subcluster and visualised via heatmap (Figure 4C). Interestingly, the Ex‐1 subcluster exhibited a marked increase in relative abundance in FCDI, constituting the largest proportion of nuclei among all subclusters (*p* < .001, Figure 4D). This subpopulation, enriched in superficial‐layer ENs, was characterised by high expression of CBLN2, CREB1L, PDGFD and HS6ST3 (Supporting Information S15), and was thus designated as *CBLN2* ^high^Ex‐1. These genes have been implicated in neurogenesis, neuronal migration, synaptic plasticity, signal transduction and blood‒brain barrier homeostasis. Mapping previously reported epilepsy‐associated genes from the BrainBase database (https://ngdc.cncb.ac.cn/brainbase), epilepsy susceptibility genes from genome‐wide association studies (GWAS)^31^, ^32^ and MCD‐related epilepsy genes^33^ onto the DEGs of each subcluster revealed significant alterations in multiple epilepsy‐related genes within the *CBLN2* ^high^Ex‐1 subcluster (Figure 4E and Supporting Information S16‒S18). Specifically, 560 genes were upregulated and 141 were downregulated in this cluster (|avg_log2FC| ≥  .1, adjusted *p*‐value < .05; Supporting Information S19). GO enrichment analysis revealed that these DEGs were involved in a broad array of neurobiological processes, including glutamatergic synapse function, neurogenesis, axon development, ion channel activity, neuronal projection development, membrane potential regulation and neuronal migration (Figure 4F and Supporting Information S20). KEGG pathway analysis further highlighted extensive signalling pathway dysregulation, including axon guidance, calcium signalling, the cGMP‒PKG pathway, glutamatergic synapses and phospholipase D signalling (Figure 4G and Supporting Information S21). To pinpoint genes implicated in cortical development and epilepsy, we extracted DEGs involved in ‘regulation of neuronal projection development’, ‘regulation of nervous system development’, ‘regulation of membrane potential’ and ‘neuronal migration’, identifying NTRK2 and FGF13 as overlapping candidates (Figure 4H). NTRK2 (TrkB), a member of the neurotrophin receptor family, binds brain‐derived neurotrophic factor (BDNF) and activates MAPK/ERK and PI3K/Akt pathways to regulate neural development and neuronal survival.^34^ FGF13, a member of the fibroblast growth factor (FGF) family, modulates voltage‐gated sodium channel function in neurons, influencing excitability and action potential generation.^35^

**FIGURE 5:**
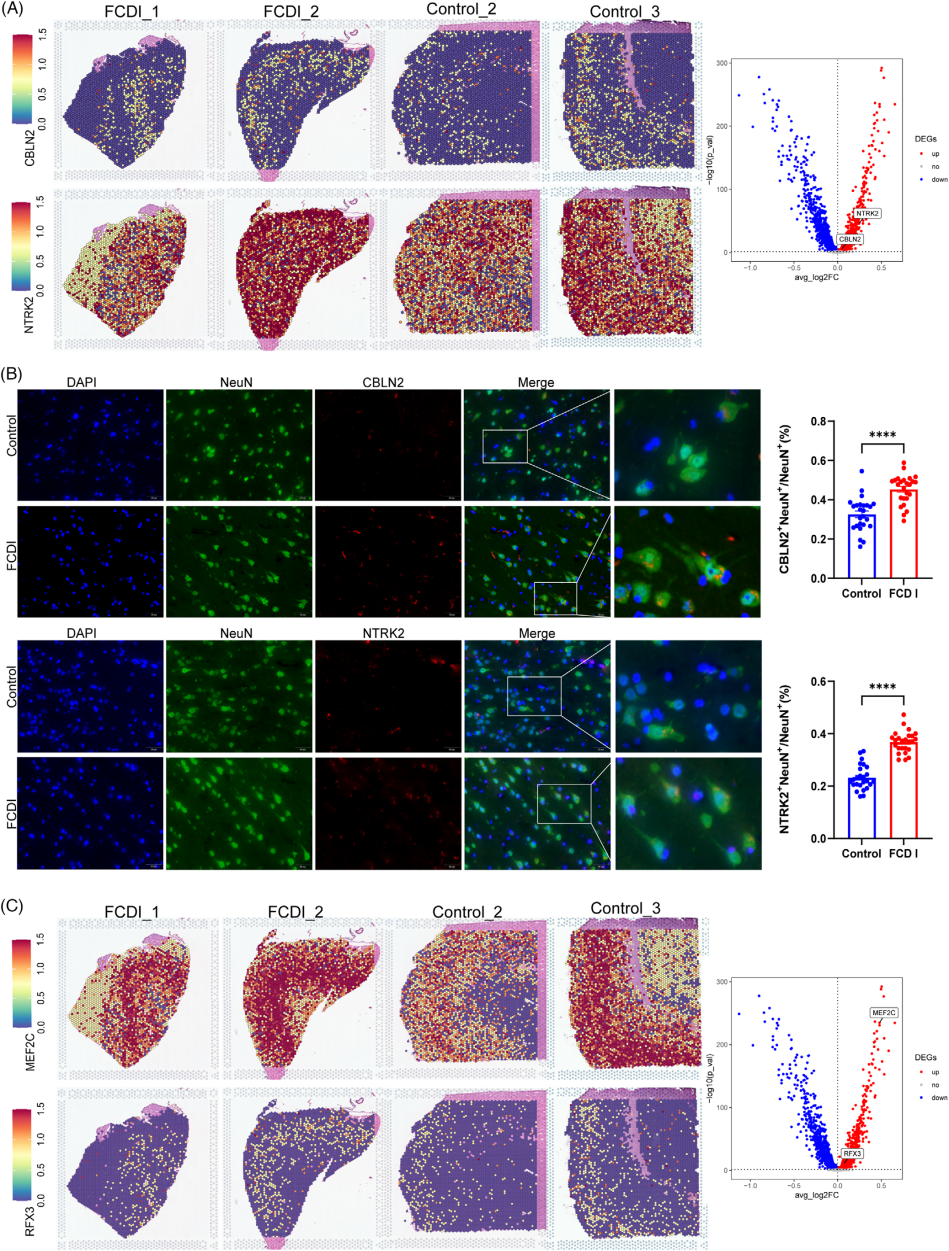
Spatial expression distribution and experimental validation of hub genes and transcription factors (TFs). (A) Spatial expression distribution of CBLN2 and NTRK2 genes. (B) Immunofluorescence (IF) experiments to validate the protein expression levels of CBLN2 and NTRK2. Scale bars = 50µm. Five fields of view were randomly selected for each sample, *n* = 5 per group. An independent *t*‐test was performed, and a two‐sided *p*‐value < .05 was considered significant. CBLN2, *t* = 5.609; NTRK2, *t* = 10.79. (C) Spatial expression distribution of the TFs MEF2C and RFX3. ^**^ *p* < .01,^***^ *p* < .001,^****^ *p* < .0001.

snRNA‐seq analysis revealed upregulation of CBLN2, NTRK2 and FGF13 in FCDI (Figure 4I). To validate these findings, we performed WB analysis on frozen brain tissue, which confirmed increased protein expression of CBLN2 and NTRK2 in FCDI patients (Figure 4J). Moreover, immunofluorescence (IF) double staining on paraffin‐embedded brain tissue sections demonstrated elevated CBLN2 and NTRK2 protein expression in ENs from FCDI patients (Figure 5B), consistent with their spatial distribution in ST‐seq data (Figure 5A). Collectively, these results validate the subtype‐specific transcriptional alterations identified by snRNA‐seq at both the transcriptome and protein levels. These findings suggest that the *CBLN2* ^high^Ex‐1 subcluster may contribute to FCDI pathogenesis, potentially via enhanced neuronal excitability and disrupted cortical development.

### 3.3 Regulatory mechanisms of Ast‐specific genes

#### 3.3.1 Increased heterogeneity of Ast in FCDI

**FIGURE 6:**
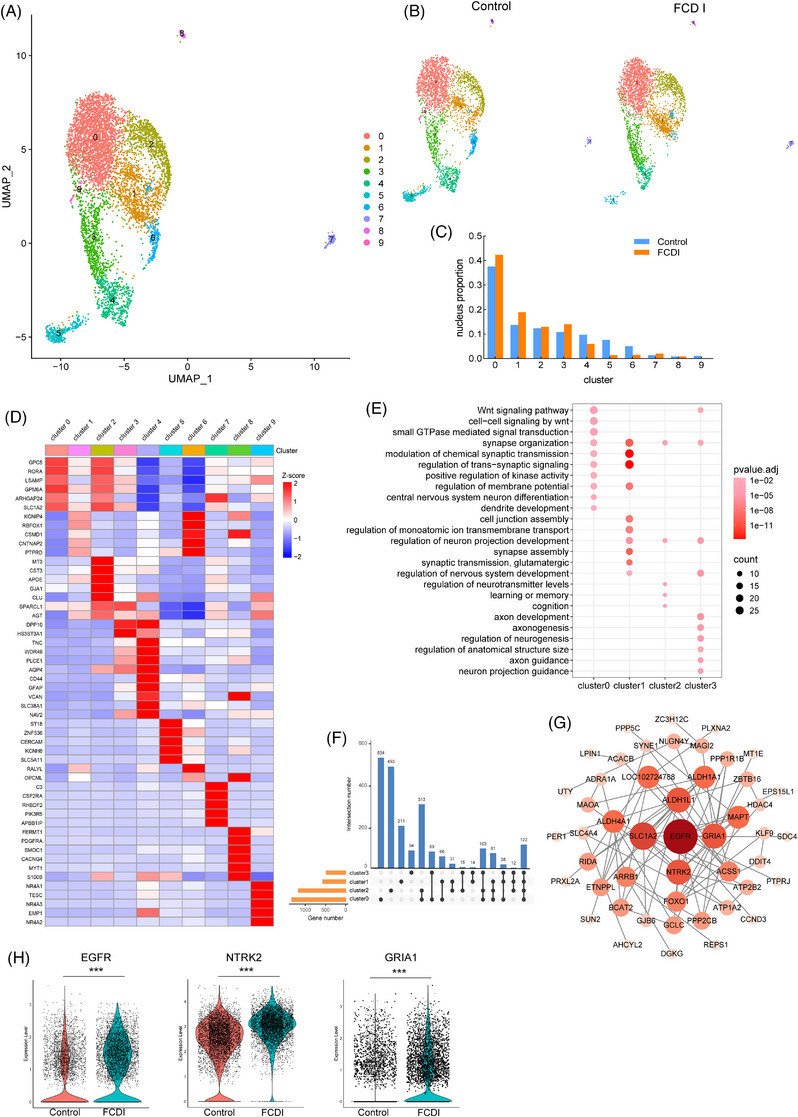
Increased heterogeneity of focal cortical dysplasia type I (FCDI) astrocytes (Ast). (A) UMAP plot of Ast after secondary dimensionality reduction clustering, with nuclei coloured by clusters. (B) UMAP plot of astrocyte subpopulations in the control and FCDI groups. (C) The proportion of different cell subgroups in the control and FCDI groups. (D) Heatmap of the top five highly expressed genes in each subpopulation. (E) The Gene Ontology (GO) functional enrichment bubble plot of differentially expressed genes (DEGs) in the Ast‐0, Ast‐1, Ast‐2 and Ast‐3 subpopulations. (F) Venn diagram of intersecting DEGs among Ast‐0, Ast‐1, Ast‐2 and Ast‐3 subpopulations. (G) Protein‒protein interaction (PPI) network of intersecting DEGs. (H) mRNA expression levels of hub genes in the control and FCDI groups. Wilcoxon rank‐sum test showed adjusted *p*‐value of 1.23E‐126 for EGFR, 9.17E‐215 for NTRK2 and 2.28E‐80 for GRIA1.^**^ *p* < .01, ^***^ *p* < .001, ^****^ *p* < .0001.

To investigate Ast‐specific alterations in FCDI, we performed secondary dimensionality reduction on Ast from snRNA‐seq data and identified 10 distinct subclusters (designated ‘Ast’; Figure 6A). Comparison of subcluster distributions between FCDI and control group revealed an increased proportion of several major Ast populations (Ast‐0, Ast‐1, Ast‐2, Ast‐3 and Ast‐7) in the FCDI group (Figure 6B,C). Each subcluster exhibited distinct transcriptional signatures (Figure 6D and Supporting Information S22). Ast‐0 showed high expression of GPC5,^36^, ^37^ RORA,^38^, ^39^ LSAMP^40^ and GPM6A,^41^ genes associated with neurodevelopment, cell adhesion and synaptic transmission. Ast‐1 was enriched for KCNIP4,^42^ RBFOX1,^43^, ^44^, ^45^ CNTNAP2^46^, ^47^ and PTPRD,^48^ which are involved in RNA splicing regulation, ion channels and neuron‒glia interactions. Ast‐2 was characterised by elevated levels of MT3, CST3, CLU, GJA1 and APOE,^27^ all of which have been associated with cell activation, stress responses and lipid metabolism. Ast‐7 highly expressed C3,^49^, ^50^ a key mediator in immune and inflammatory responses. These findings indicate increased Ast activation and enhanced cellular heterogeneity in FCDI. GO enrichment analysis of the DEGs highly expressed in these subclusters revealed their involvement in Wnt signalling, small GTPase‐mediated signal transduction, synaptic transmission, membrane potential regulation, ion transmembrane transport, neurotransmitter level modulation and learning and memory processes (Figure 6E and Supporting Information S23). We profiled the DEGs in each Ast subcluster and identified 122 genes commonly shared in Ast‐0, Ast‐1, Ast‐2 and Ast‐3 (Figure 6F). To further elucidate regulatory interactions, we constructed a PPI network using Cytoscape (Figure 6G). This analysis identified EGFR, NTRK2 and GRIA1 as hub genes upregulated in FCDI (Figure 6H). EGFR, a receptor tyrosine kinase, regulates cell proliferation, differentiation and survival.^51^ GRIA1, a subunit of AMPA‐type glutamate receptors, plays a crucial role in excitatory synaptic transmission.^52^

#### 3.3.2 Ast maturation and differentiation are impaired in FCDI

**FIGURE 7:**
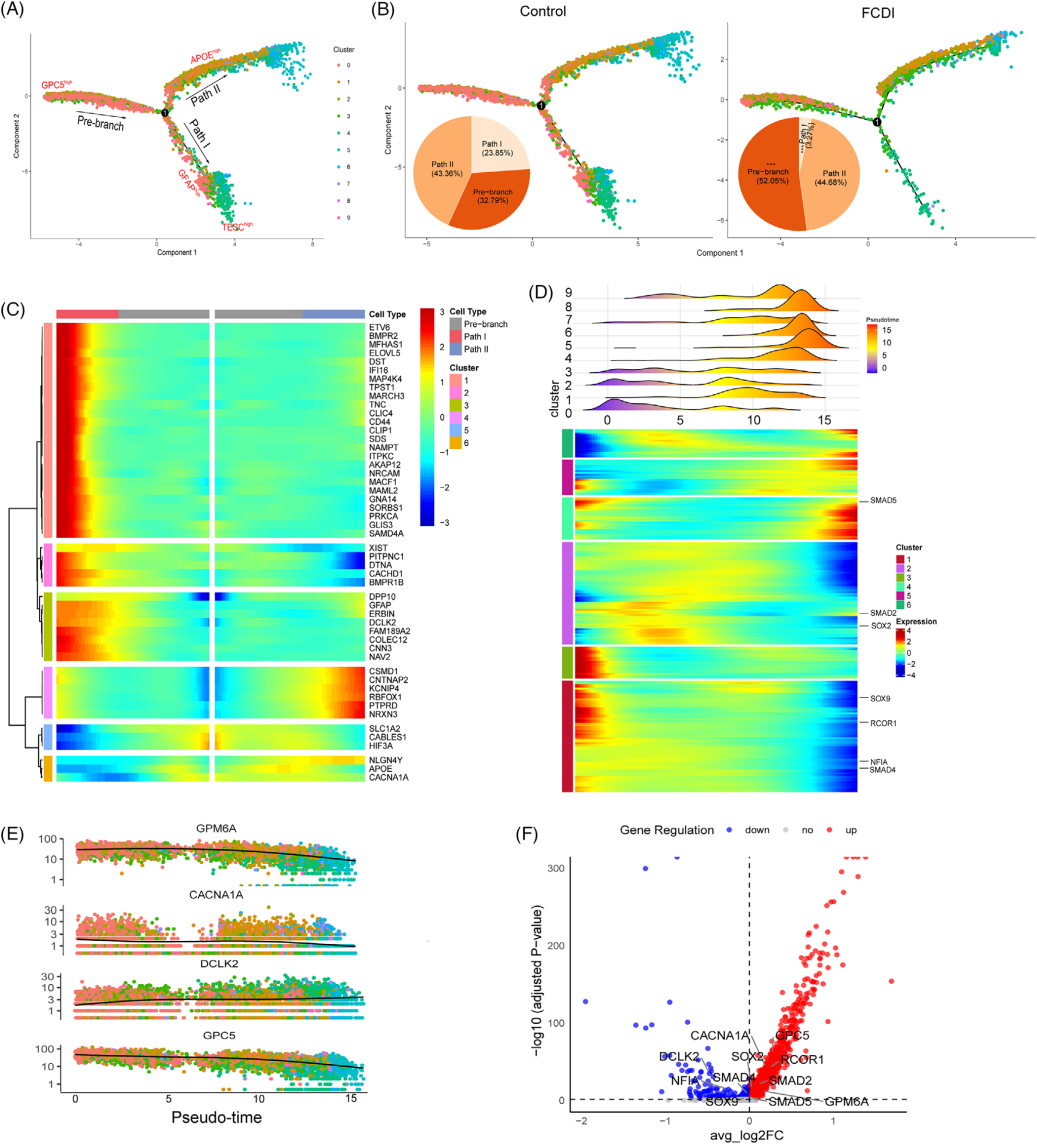
Pseudotime analysis of astrocytes. (A) Pseudotime trajectory reconstruction of astrocytes showing three trajectory states, including a Pre‐branch state and two paths (Path I and Path II). Cells are coloured by cluster, and selected marker genes enriched along different states are highlighted. (B) Proportion of cell nuclei in different differentiation states between the focal cortical dysplasia type I (FCDI) and control groups. A chi‐square test was performed, and a two‐sided *p*‐value < .05 was considered significant. Pre‐branch, *χ* ^2^ = 141.74; Path I, *χ* ^2^ = 495.85. ^***^ *p* < .001. (C) Heatmap showing the correlation between differentially expressed genes (DEGs) and cell different trajectory states. The centre of the plot represents the Pre‐branch, while the left and right sides correspond to Path I and Path II, respectively. The *y*‐axis labels indicate genes, and the colour represents gene expression levels. (D) The distribution of cell subpopulations along the pseudotime (ridge plot, top) and the expression level changes of predicted transcription factors along the pseudotime (heatmap, bottom). The *y*‐axis labels indicate genes, and the colour represents gene expression levels. (E) Changes in the expression of GPM6A, CACNA1A, DCLK2 and GPC5 genes along the pseudotime trajectory. (F) The volcano plot shows the differential expression of certain genes and transcription factors (TFs) between the FCDI and control groups. Upregulated genes are represented by red dots, downregulated genes by blue dots, and genes with no significant change are shown as grey dots.

To further elucidate the molecular mechanisms driving the abnormal Ast activation and heterogeneity in FCDI, we performed pseudotemporal trajectory analysis of Ast subpopulations using Monocle2 on snRNA‐seq data. Based on the developmental stages of different cell types, Ast from both the FCDI and control groups were accurately positioned on the inferred trajectory (Figure S9). According to their distribution along trajectory branches, Ast were classified into three states: Pre‐branch, Path I and Path II (Figure 7A). In the control group, 32.79% of nuclei were classified as Pre‐branch, 23.85% as Path I and 43.36% as Path II. In contrast, the FCDI group exhibited a shifted distribution, with an increased proportion of Pre‐branch nuclei (52.05%) and a marked reduction in Path I (3.27%), while Path II remained comparable between groups (44.68%). Consistently, Path I was significantly more abundant in controls (*p* < .001), whereas the Pre‐branch population was significantly enriched in the FCDI group (*p* < .001); no significant difference was observed for Path II (*p* >  .05) (Figure 7B and Supporting Information S24). This altered trajectory pattern may suggest a potential developmental blockade or delay in Ast differentiation in FCDI, which could be associated with an impaired transition towards mature functional states. Gene expression profiling across differentiation states revealed that cells in the Pre‐branch predominantly expressed GPC5, a gene implicated in glial development and maintenance of undifferentiated cell identity. Cells in the Path I showed enriched expression of GFAP and TESC, markers of maturing Ast. However, the drastic reduction in Path I cells in FCDI indicates a disruption in the astrocytic maturation process. Cells in the Path II branch exhibited elevated APOE expression, associated with mature Ast function, particularly in lipid metabolism and neural repair. These findings suggest that Ast differentiation trajectories in FCDI are dysregulated, likely due to impaired signalling mechanisms, resulting in a failure to activate maturation programs critical for astrocytic function.

To further explore this phenomenon, we constructed a dynamic heatmap of DEGs before and after key differentiation nodes along the trajectory (Figure 7C and Supporting Information S25). Observing different time points along the trajectory revealed changes in gene expression, with ion channel‐related genes (CACNA1A and KCNIP4), ion transport and cell homeostasis‐related genes (SLC1A2 and CLIC4), cell proliferation and signalling‐related genes (MAP4K4, PRKCA, CABLES1, MAML2 and CSMD1), neurodevelopment‐related genes (GNA14, RBFOX1 and SAMD4A) and cytoskeletal and structural stability genes (AKAP12 and GFAP) exhibiting notable alterations in the FCDI group. To identify TFs regulating these DEGs, we utilised pySCENIC analysis on Ast DEGs from the snRNA‐seq data and identified several TFs with dynamic expression patterns along the pseudotemporal trajectory, shown in heatmaps (Figure 7D,F and Supporting Information S26 and S27). Among these, the differentially expressed TFs SMAD2, SMAD4, SMAD5, SOX2, SOX9, RCOR1 and NFIA are likely key regulators of Ast differentiation. Additionally, we observed that GPM6A, CACNA1A and GPC5 exhibited a gradual decline in expression along the normal differentiation trajectory (Figure 7E), but their expression levels were markedly elevated in the FCDI group (Figure 7F). GPM6A, a signalling transducer responsive to extracellular signals, promotes lipid raft aggregation and related signalling molecules, thereby accelerating the determination of neuronal polarity.^41^ In contrast, DCLK2 showed an upregulation trend along the normal trajectory (Figure 7E), but its expression was significantly reduced in the FCDI (Figure 7F). DCLK2, along with its family member DCX, participates in hippocampal structure formation, and its deletion results in severe epileptic phenotypes and lethality.^53^ Overall, these findings suggest that Ast in the FCDI group exhibit an aberrant differentiation pattern, characterised by an increased number of undifferentiated Ast and a reduced number of mature Ast. Furthermore, we identify potential key TFs that may regulate this abnormal differentiation process, offering insights for understanding the pathogenesis of FCDI.

### 3.4 Characteristics of cell‒cell communication between ENs and Ast in FCDI

#### 3.4.1 Overall alterations in intercellular communication

**FIGURE 8:**
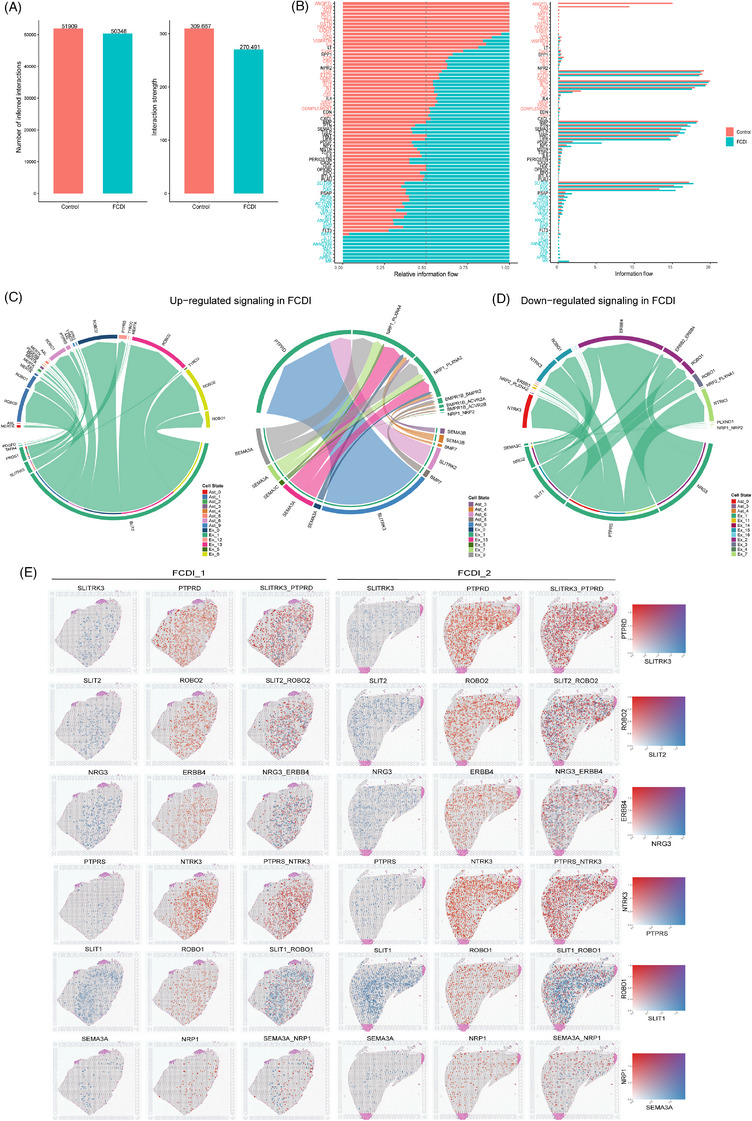
Aberrant communication between excitatory neurons and astrocytes in focal cortical dysplasia type I (FCDI). (A) Statistical analysis of the overall number and strength of cell communication between excitatory neurons and astrocytes in the FCDI and control groups. (B) Overall changes in signalling pathways of cell communication between excitatory neurons and astrocytes in the FCDI and control groups. The *y*‐axis represents signalling pathways, with red indicating increased communication in the control group, green indicating increased communication in the FCDI group, and black indicating no statistically significant difference between the two groups. (C) The chord diagram shows the upregulated signalling pathway ligand‒receptor pairs in the FCDI group, with *CBLN2* ^high^Ex‐1 acting as the source (left) and target (right). (D) The chord diagram shows the downregulated signalling pathway ligand‒receptor pairs in the FCDI group, with *CBLN2* ^high^Ex‐1 acting as the source. The outer circle represents the interacting cell types, with arrows pointing from the source cells to the target cells. The inner circle represents the intensity of interactions received by the target cells, with the size of the inner bars proportional to the strength of the interaction received by the target cells. (E) Spatial co‐localisation of significantly altered ligand‒receptor pairs in FCDI. The blue dots in the figure represent the spatial distribution of ligands, the red dots represent the spatial distribution of receptors, and the overlapping area of the two appears purple.

Aberrant communication between ENs and Ast has been increasingly implicated in disrupted cortical circuit development and various neurological disorders. Compared to controls, the FCDI group exhibited a reduced number of predicted intercellular interactions, yet an overall increase in communication strength (Figure 8A). Pathway‐level analysis further revealed substantial dysregulation in multiple signalling cascades. Specifically, pathways such as PTN, EGF, platelet‐derived growth factor (PDGF), ACTIVIN, CCK, SLITRK, GAS, ANGPT, WNT, ncWNT, SEMA3, PSAP and BAFF were upregulated in the FCDI, whereas signalling through LIFR, ANGPTL and CCL was notably downregulated (Figures 8B and S10 and Supporting Information S28 and S29). Pleiotrophin (PTN), primarily secreted by Ast, was inferred to potentially signal to both Ast and ENs. Previous studies have shown that PTN can activate AKT signalling via its receptor PTPRZ1 and is involved in adult neurogenesis and neural plasticity.^54^ Similarly, PDGF is also mainly produced by Ast and influences both Ast and ENs through PDGFR‐mediated signalling. PDGF signalling has been reported to be associated with neurogenesis, cell proliferation and survival, synaptogenesis, ligand‐gated ion channel activity and neuronal development, while excessive activation of PDGF signalling in Ast has been shown to impair the blood‒brain barrier and exacerbate neuroinflammation.^55^, ^56^ Cholecystokinin (CCK), initially identified as a marker of inhibitory neurons, is also expressed in glutamatergic neurons. In FCDI, CCK is secreted by ENs and may be involved in neuron‒neuron interactions associated with neuronal activity and synaptic plasticity. CCK signalling has been reported to enhance excitatory long‐term potentiation (eLTP) at CCK‐positive glutamatergic synapses and has been implicated in epileptogenic processes.^57^, ^58^ SLIT and NTRK‐like family member (SLITRK), generated by ENs and targeting both ENs and Ast, has been reported to interact with LAR‐receptor‐type protein tyrosine phosphatase (RPTP) family members and has been implicated in neuronal growth and synaptogenesis, contributing to excitatory‒inhibitory balance.^59^, ^60^ WNT signalling was predicted to originate primarily from ENs and to potentially signal to both ENs and Ast, and it has been closely associated with neocortical development.^61^, ^62^ Aberrant activation of the Wnt/β‐catenin signalling pathway may be associated with epileptogenesis through abnormal neurogenesis, hyperexcitability, and neuroinflammation.^63^, ^64^

#### 3.4.2 Unique interactions between *CBLN2* ^high^Ex‐1 ENs and Ast

We further inferred the cell‒cell communication between the *CBLN2* ^high^Ex‐1 and other EN and Ast subclusters. In the FCDI group, aberrant ligand‒receptor interactions were predicted across multiple signalling pathways (Supporting Information S30 and S31). Notably, several ligand‒receptor pairs were significantly upregulated (Figure 8C), including SEMA3‒NRP1, SLITRK3‒PTPRD and SLIT2‒ROBO2, while others such as NRG3‒ERBB4, PTPRS‒NTRK3 and SLIT1‒ROBO1 were downregulated (Figure 8D). Given that snRNA‐seq lacks spatial resolution, some ligand‒receptor pairs may represent transcriptional co‐expression between cells that are not in physical proximity and thus unlikely to interact in vivo. To evaluate their potential for functional interaction, we mapped the spatial distribution of these aberrant ligand‒receptor pairs. Spatial transcriptomics revealed that these pairs exhibit co‐localised expression patterns within the tissue microenvironment, primarily localised in layers II‒IV of the cortex (Figure 8E), suggesting spatial permissiveness for potential signalling interactions.

SEMA3A is a chemotropic guidance cue that has been shown to regulate neuronal polarity through its interaction with NRP1 and has been implicated in neurodevelopmental processes.^65^ Dysregulated SEMA3A expression is associated with abnormal axonal and dendritic development and has been implicated in neurodevelopmental disorders.^66^ In addition, upregulation of SEMA3A can activate Ast and contribute to glial scar formation, hindering axonal regeneration after brain injury. It also exerts anti‐angiogenic effects in pathological contexts, interfering with normal vascular development.^67^ PTPRD, a member of the RPTP family, is a transmembrane molecule involved in diverse cellular processes.^68^ Presynaptic PTPRD can interact with the postsynaptic adhesion molecule SLITRK3 and may be involved in the regulation of glutamatergic and GABAergic synapse development, which has been associated with excitatory‒inhibitory synaptic balance.^69^ SLIT2 is a well‐characterised axon guidance molecule that signals through its receptor ROBO2 to regulate radial neuronal migration, likely by influencing neuronal orientation and polarity.^70^ The NRG3‒ERBB4 axis has been implicated in brain development and synaptic regulation, and although its upregulation has been associated with suppression of epileptiform activity,^71^, ^72^ it was predicted to be downregulated in FCDI. PTPRS and NTRK3 have been implicated in neuronal autophagy and plasticity, axon guidance and synaptic innervation in cortical regions,^73^ although the mechanisms underlying their interaction remain unclear.^74^ The SLIT1‒ROBO1 axis may modulate cellular growth, differentiation and migration through downstream signalling and is involved in cortical neurogenesis and angiogenesis during brain development.^75^

## 4 DISCUSSION

**FIGURE 9:**
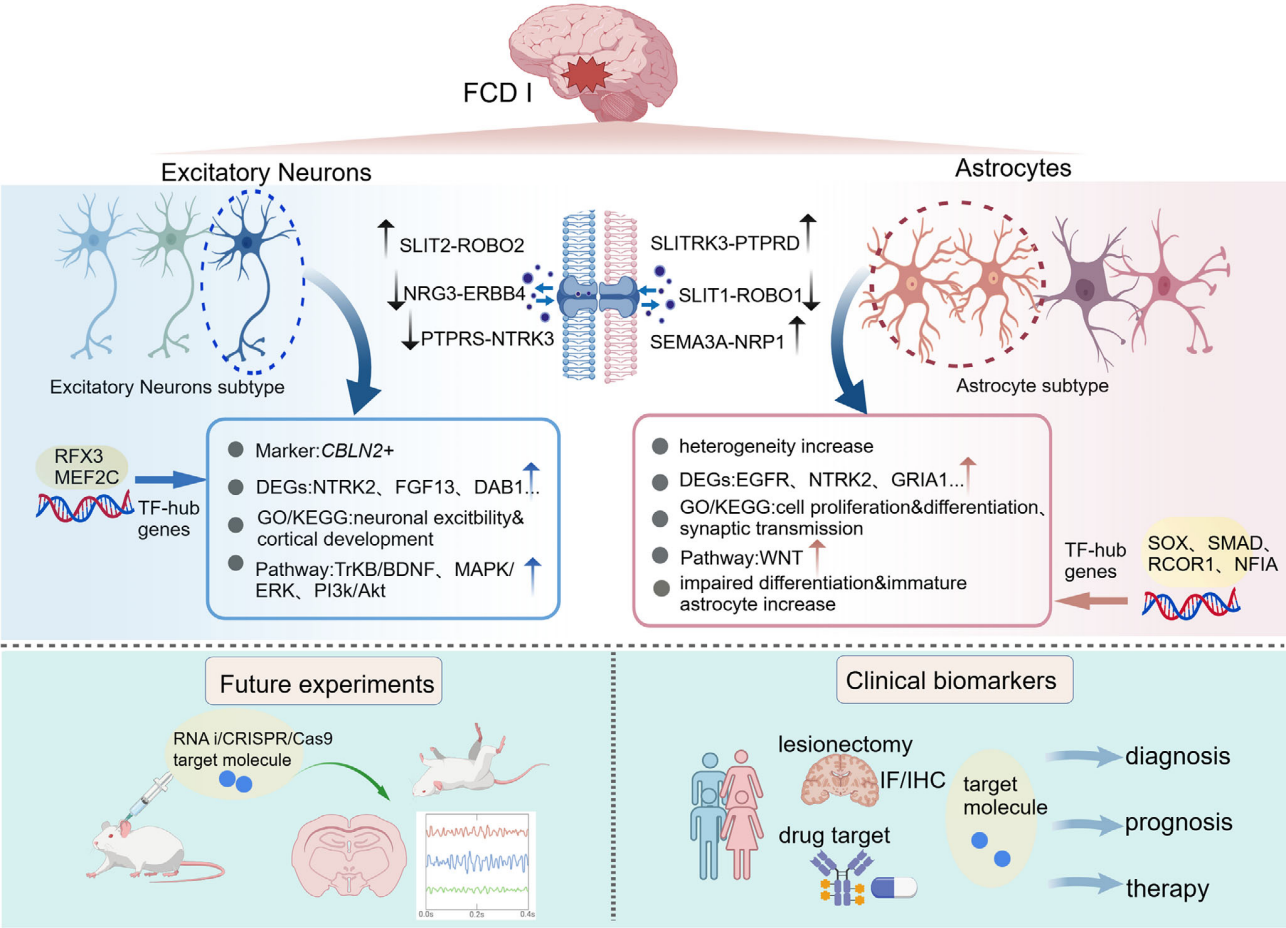
Summary of the molecular signatures and future directions in focal cortical dysplasia type I (FCDI).

In this study, we present the first integrative analysis of snRNA‐seq and ST‐seq from matched samples to elucidate the cell‐type‐specific transcriptional landscape and spatial architecture of epileptogenic cortex in FCDI. Our findings highlight ENs and Ast as key cellular contributors to FCDI pathogenesis. Deep profiling of ENs identified disease‐associated hub genes, characterised their spatial distribution and enabled the construction of a TF‒hub gene regulatory network. Notably, we discovered a distinct subcluster of ENs, *CBLN2* ^high^Ex‐1, which may be involved in epileptogenic processes. Ast analysis revealed pronounced heterogeneity in the FCDI group, with upregulation of genes linked to proliferation and ion channel activity. Furthermore, pseudotime trajectory analysis uncovered disrupted differentiation dynamics in Ast, characterised by an accumulation of immature cells. We predicted candidate TFs potentially regulating this aberrant differentiation process. These findings were further validated at the protein level, corroborating the dysregulated expression of key genes and TFs. Finally, we identified multiple aberrant signalling pathways mediating communication between ENs and Ast, with spatial mapping confirming the co‐localisation of ligand‒receptor pairs. These results suggest that impaired neuron‒Ast crosstalk may be associated with cortical remodelling and epileptogenic susceptibility in FCDI (Figure 9 and Table 1).

**TABLE 1:** Clinically oriented summary of key disease‐associated markers.

Gene/protein	Cell type/state	Direction and avg_log2FC	Biological role	Clinical relevance	Evidence level
CBLN2	Excitatory neurons	↑.82	Synapse assembly and excitatory synaptic modulation	Potential biomarker	snRNA‐seq and protein
NTRK2	Excitatory neurons	↑.35	Synaptic transmission and membrane potential	Drug target (Trk inhibitors available)	snRNA‐seq and protein
FGF13	Excitatory neurons	↑.16	Neuronal polarisation and migration	Biomarker	snRNA‐seq
DAB1	Excitatory neurons	↑.17	Neuronal migration	Biomarker	snRNA‐seq
RFX3	Excitatory neurons	↑.1	Transcriptional regulation and neuronal migration	Biomarker	snRNA‐seq and protein
MEF2C	Excitatory neurons	↑.29	Developmental growth and glutamatergic synaptic transmission	Biomarker/prognosis	snRNA‐seq and protein
EGFR	Astrocytes	↑.57	Cell proliferation, differentiation and survival	Drug target (FDA‐approved inhibitors)	snRNA‐seq
GRIA1	Astrocytes	↑.56	Postsynaptic density and synaptic transmission	Therapeutic target	snRNA‐seq
NFIA	Astrocytes	↓.25	Transcriptional regulation of gliogenesis and neurodevelopment	Biomarker	snRNA‐seq
RCOR1	Astrocytes	↑.23	Transcriptional repression and neurodevelopment	Potential biomarker	snRNA‐seq

Abbreviation: snRNA‐seq, single‐nucleus RNA sequencing.

Our single‐nucleus and spatial transcriptomic analyses revealed aberrant activity of ENs and Ast in FCDI, accompanied by a marked increase in cell density within cortical layers II‒III, which may be associated with the pathological alterations in FCDI. In ENs, we identified a hub gene module highly associated with FCDI, primarily involved in neuronal development, migration and synaptic function. Key genes implicated in neuronal development and migration include DAB1, a critical component of the Reelin signalling pathway that regulates neuronal laminar positioning and whose dysregulation has been linked to cortical architectural disorganisation.^76^, ^77^, ^78^ SATB2, a chromatin remodeller, plays a pivotal role in the specification and axonal targeting of cortical projection neurons.^79^, ^80^ CUX2 encodes a TF essential for the differentiation and dendritic morphogenesis of upper‐layer ENs.^81^ Regarding synaptic signalling and ion channel regulation, GRIA4 encodes subunit 4 of the AMPA‐type glutamate receptor, which mediates fast excitatory synaptic transmission and plasticity.^26^, ^82^ CBLN2, a secreted glycoprotein, modulates the formation and stabilisation of glutamatergic synapses,^83^, ^84^ thereby influencing excitatory signalling. KCNIP4 regulates Kv4 potassium channel activity and action potential repolarisation.^85^ HS6ST3, a heparan sulphate sulfotransferase, may influence ion channel function by modifying ligand accessibility, such as for FGFs.^86^, ^87^ Collectively, the dysregulated expression of these genes may converge to drive both cortical structural disorganisation and enhanced neuronal excitability in FCDI.

Transcriptional regulatory analysis of the hub gene module revealed that the TFs (MEF2C and RFX3) regulate the expression of multiple hub genes. MEF2C is predominantly expressed in cortical ENs and subsets of inhibitory interneurons,^88^ where it orchestrates neurogenesis, neuronal differentiation, survival, synapse development and plasticity within cortical circuits.^89^, ^90^ GWAS have identified MEF2C as a common genetic risk factor for various neurodevelopmental and neuropsychiatric disorders. Microdeletions or pathogenic coding variants in MEF2C cause MEF2C haploinsufficiency syndrome, characterised by intellectual disability and epilepsy.^91^ Notably, embryonic deletion of MEF2C in ENs does not markedly affect cortical structure, whereas its postnatal overexpression in newly generated neurons leads to displaced hippocampus.^92^ RFX3, a member of the regulatory factor X family, modulates gene expression by binding specific DNA motifs and plays a role in cell differentiation and development. It is also involved in regulating genes essential for ciliogenesis, thereby influencing neuronal migration and positioning.^93^ A recent whole‐exome sequencing study by Torio et al. reported a de novo heterozygous pathogenic variant in RFX3 in a patient with infantile spasms and autism, further implicating its role in early‐onset neurodevelopmental disorders.^94^ Strikingly, these core regulatory genes exhibit the most pronounced differential expression within the *CBLN2* ^high^Ex‐1 subcluster, suggesting that this excitatory neuronal subset may represent a key cellular node in FCDI pathophysiology.

Previous studies have suggested that certain single‐neuron epileptogenic factors may contribute to specific brain malformations by altering the excitatory/inhibitory (E/I) ratio.^95^ Notably, we found that the *CBLN2* ^high^Ex‐1 subcluster harbors a large number of DEGs previously implicated in FCD and epilepsy, suggesting that this neuronal subset may represent a core cellular hub within the epileptogenic network. CBLN2, a member of the cerebellin family of secreted proteins, is selectively expressed in the cerebral cortex and mediates the assembly of trans‐synaptic adhesion complexes.^96^, ^97^ Upregulation of CBLN2 has been shown to increase dendritic spine density in cortical neurons.^83^ At hippocampal synapses, NRXN activates the CBLN2‒GluD1 complex, selectively enhancing NMDAR‐mediated excitatory postsynaptic currents (EPSCs) while suppressing AMPAR‐mediated EPSCs, thereby modulating synaptic excitability.^84^, ^98^ Recent studies have also shown that CBLN2 plays important roles in regulating panic‐like defensive behaviours^99^ and mediating neuropathic pain^100^ in mice. However, its function in cortical development‐related epilepsy has not been fully explored. In our data, within the *CBLN2* ^high^Ex‐1, the hub genes NTRK2 and FGF13 were both upregulated in FCDI and enriched in biological processes including regulation of neuronal projection development, nervous system development, membrane potential regulation and neuronal migration. Consistently, WB and IF confirmed increased protein levels of CBLN2 and NTRK2 in FCDI tissue. FGF13, a microtubule‐stabilising protein from the FGF family, is expressed in cortical neurons during development and regulates neuronal polarisation and migration.^101^ Elevated expression of FGF13 in dysplastic cortical regions of FCD patients has been associated with impaired neuronal polarity, disorganised cortical lamination and aberrant network formation.^102^ NTRK2 encodes TrkB, the high‐affinity receptor for BDNF.^103^ A recurrent NTRK2 variant (c.1301A > G, p. Tyr434Cys) has been shown to increase TrkB activity, resulting in severe developmental delay, intractable epilepsy, visual deficits and feeding difficulties.^104^ During seizures, BDNF expression is markedly upregulated, activating TrkB‐mediated MAPK, PI3K/Akt and PLCγ signalling pathways,^105^ as well as adenosine/A2AR retrograde signalling,^106^ collectively enhancing excitatory synaptic transmission while dampening inhibitory inputs. These changes promote hypersynchronous neuronal activity and seizure network formation. Inhibition of the BDNF/TrkB signalling axis has been shown to reduce neuronal hyperexcitability and neuroinflammation, mitigating seizures in both temporal lobe epilepsy and post‐traumatic epilepsy models.^34^, ^107^ However, the precise role of TrkB signalling in FCD remains to be fully elucidated. Together, these findings suggest that the *CBLN2* ^high^Ex‐1 may contribute to FCDI pathogenesis by modulating both cortical development and neuronal excitability.

Ast are a diverse and heterogeneous population of glial cells whose number and complexity increase with the development of the central nervous system.^108^ Over the past two decades, synaptic transmission has increasingly been recognised as a process not exclusive to neurons. The concept of the ‘tripartite synapse’ has gained traction, which encompasses astrocytic processes interacting with presynaptic and postsynaptic neuronal membranes, underscoring the active role of Ast in shaping the structural and functional synaptic microenvironment.^109^, ^110^ Recent studies have further highlighted astrocytic functional diversity. For instance, de Ceglia et al. identified a hippocampal Ast subpopulation expressing neuronal synaptic transcripts and a glutamatergic‐like vesicular release mechanism, termed ‘glutamatergic astrocytes’.^111^ Moreover, activated Ast release ATP/ADP and glutamate via vesicular exocytosis, with ATP/ADP acting on microglia to amplify glial transmission.^112^ In FCDI, we observed increased heterogeneity and abnormal activation of Ast subpopulations, which were implicated in neurodevelopment, cell adhesion, ion channel regulation, inflammation, lipid metabolism and neuron‒glia interactions. Hub genes such as EGFR, GRIA1 and NTRK2 were upregulated in Ast from FCDI patients, suggesting that Ast may contribute to FCDI pathogenesis through the regulation of cell proliferation, AMPA receptor signalling and neurotrophic modulation. Ast are also known to IL‐17 and TrkB expression during neuroinflammatory responses.^113^ These findings align with emerging evidence of Ast as regulators of neuronal homeostasis, excitability, synaptic plasticity and neuroinflammation.

Proper differentiation of Ast is essential for normal development and function of the nervous system. Compared to controls, Ast in FCDI patients exhibited marked differentiation defects, characterised by a higher proportion of cells remaining in an undifferentiated state and a reduced fraction progressing towards maturation. These immature astrocytic populations may contribute to the epileptogenic potential of FCDI. Ast integrate into neural circuits by responding to neuronal activity via neurotransmitter receptors, ion channels and metabolite transporters.^114^ We found that CACNA1A, which encodes the P/Q‐type voltage‐gated calcium channel (Cav2.1), was significantly upregulated in FCDI. Overexpression of Cav2.1 may enhance calcium influx in Ast, triggering aberrant calcium oscillations and promoting gliotransmitter (glutamate) release, thereby directly activating surrounding neurons. This glutamate, released via calcium‐dependent exocytosis, may lead to NMDA receptor overactivation in neurons and epileptiform plasticity, such as LTP‐like hyperexcitability.^115^, ^116^, ^117^

Mechanisms regulating Ast differentiation involve intracellular and extracellular signalling, dynamic TF expression, and epigenetic regulation.^108^ We predicted several TFs that may govern the dysregulated differentiation programs, including members of the SMAD family (SMAD2, SMAD4 and SMAD5),^118^, ^119^, ^120^ SOX family (SOX2 and SOX9),^121^ RCOR1^122^ and NFIA,^123^ which are closely linked to TGF‐β/SMAD, JAK‐STAT and Notch signalling pathways. Taken together, Ast in FCDI exhibit increased heterogeneity, impaired differentiation, and a depletion of mature Ast populations. Ion channel imbalance and aberrant gliotransmission further exacerbate neuronal hyperexcitability and epileptiform synaptic plasticity. Dysregulation of key TFs and signalling pathways may underlie these alterations, representing critical mechanisms in FCDI pathophysiology.

Emerging studies have underscored the critical role of neuron‒glia crosstalk in cortical circuit development and disease pathogenesis.^124^, ^125^ In our analysis, multiple signalling pathways were significantly altered in the FCDI group, with aberrant activation observed in WNT, SEMA3, glial cell line‐derived neurotrophic factor (GDNF) and BAFF signalling. Notably, excessive activation of BMP and WNT pathways in FCD organoids and patient cortical tissues has been implicated in ectopic cardiomyocyte‐like cell displacement, potentially contributing to the molecular basis of cortical lamination defects.^126^ SEMA3 signalling plays an essential role in neuronal guidance, axon regeneration and cortical circuit formation^67^; its dysregulation may disrupt proper neuronal connectivity. Upregulation of GDNF may reflect a compensatory neuroprotective mechanism.^127^ In contrast, PDGF and BAFF signalling have been shown to promote epileptogenic network formation by enhancing pro‐inflammatory responses.^55^, ^56^, ^128^ We inferred that these pathways have been associated with cortical development, neuronal activity, gliotrophic support, angiogenesis, blood‒brain barrier regulation and neuroinflammation, and may be linked to both pathogenic and protective processes in FCDI. Furthermore, the *CBLN2* ^high^Ex‐1 was significantly involved in altered intercellular communication through dysregulated ligand‒receptor interactions, many of which were spatially co‐localised, indicating potential functional relevance. These changes converge on biological processes including neuronal migration, dendritic development, axonal projection, synaptic density regulation, astrocytic scar formation, excitation‒inhibition balance and neuroinflammatory responses. Such widespread alterations likely contribute to cortical mislamination and aberrant network assembly, underlying both the structural pathology and epileptogenicity observed in FCDI. Although these analyses provide valuable insights into candidate signalling pathways and potential intercellular interactions associated with FCDI, future studies incorporating longitudinal or dynamic data,^129^, ^130^ together with RNA velocity or lineage‐anchoring metrics, will be required to establish directionality and causality and may reflect more complex and dynamic patterns.^131^, ^132^

This study has several limitations. First, due to the scarcity of clinical samples and the large size of neurons, traditional scRNA‐seq methods were not suitable for effective cell isolation and transcriptomic profiling. To address this limitation, we employed snRNA‐seq, which enables transcriptomic analysis through nuclear RNA extraction. Although this approach allows robust capture of nuclear transcripts, it may exclude cytoplasmic mRNAs, potentially limiting the detection of certain genes and affecting cell‐type resolution. Nevertheless, we identified thousands of DEGs across well‐defined brain cell populations. Second, in patients with FCDI, we did not perform a direct comparison between neurons in the lesional cortex and those in the adjacent normal‐appearing cortex. Third, the trajectory and pseudotime analyses presented in this study are based on computational inference and do not represent experimentally validated temporal dynamics or causal state transitions. Moreover, spatial transcriptomic data capture static molecular states, limiting direct inference of temporal progression. Finally, future studies incorporating perturbation experiments (e.g., ligand/receptor blocking or gene knockdown/overexpression), as well as electrophysiological assessments, will be necessary to verify the functional roles of the predicted signalling pathways and their potential roles in disease pathogenesis.

## 5 CONCLUSIONS

In this research, we present the first single‐cell and spatial transcriptomic characterisation of the epileptogenic cortex in FCDI, revealing extensive transcriptional alterations and highlighting cellular and regional heterogeneity. Dysregulation of ENs and Ast, along with aberrant intercellular communication, may play critical roles in FCDI pathogenesis. These findings provide novel insights into the molecular underpinnings of FCDI and suggest potential targets for the development of precision therapies and disease‐modifying interventions.

## AUTHOR CONTRIBUTIONS

Liemin Zhou, Yaqian Zhang, Lisen Sui and Dezhi Cao conceived the study and participated in its design. Yaqian Zhang acquired and analysed the data. Qihang Zou carried out most of the experiments. Yingying Liu carried out part of the experiments. Yinchao Li and Yubao Fang drafted a significant portion of the manuscript. Tiancai Huang substantively revised the manuscript. Jiabin Yu helped to collect subject information. All the authors read and approved the final manuscript.

## CONFLICT OF INTEREST STATEMENT

The authors declare they have no conflicts of interest.

## ETHICS STATEMENT

This study follows the principles of the Declaration of Helsinki. Approval was granted by the Medical Ethics Committee of the Seventh Affiliated Hospital of Sun Yat‐Sen University (approval no. KY‐2023‐019‐02). All the participants provided written informed consent prior to inclusion in the study.

## Supporting information



Supporting Information

Supporting Information

## Data Availability

The RNA‐seq and ST‐seq datasets (FASTQ files) and processed data during the current study were deposited in GEO under accession numbers GSE295236 and GSE295237.
