# Supplementary material for: Excitatory neurons and astrocytes‐specific dysregulation and aberrant interactions are vulnerable to FCDI as suggested by single‐cell spatial transcriptomics
Source: Clin Transl Med. 2026 May 1;16(5):e70673. doi: 10.1002/ctm2.70673 (PMC13135119; doi:10.1002/ctm2.70673)
Supplement: Supplementary file 1 — Supporting Information [file CTM2-16-e70673-s002.docx]

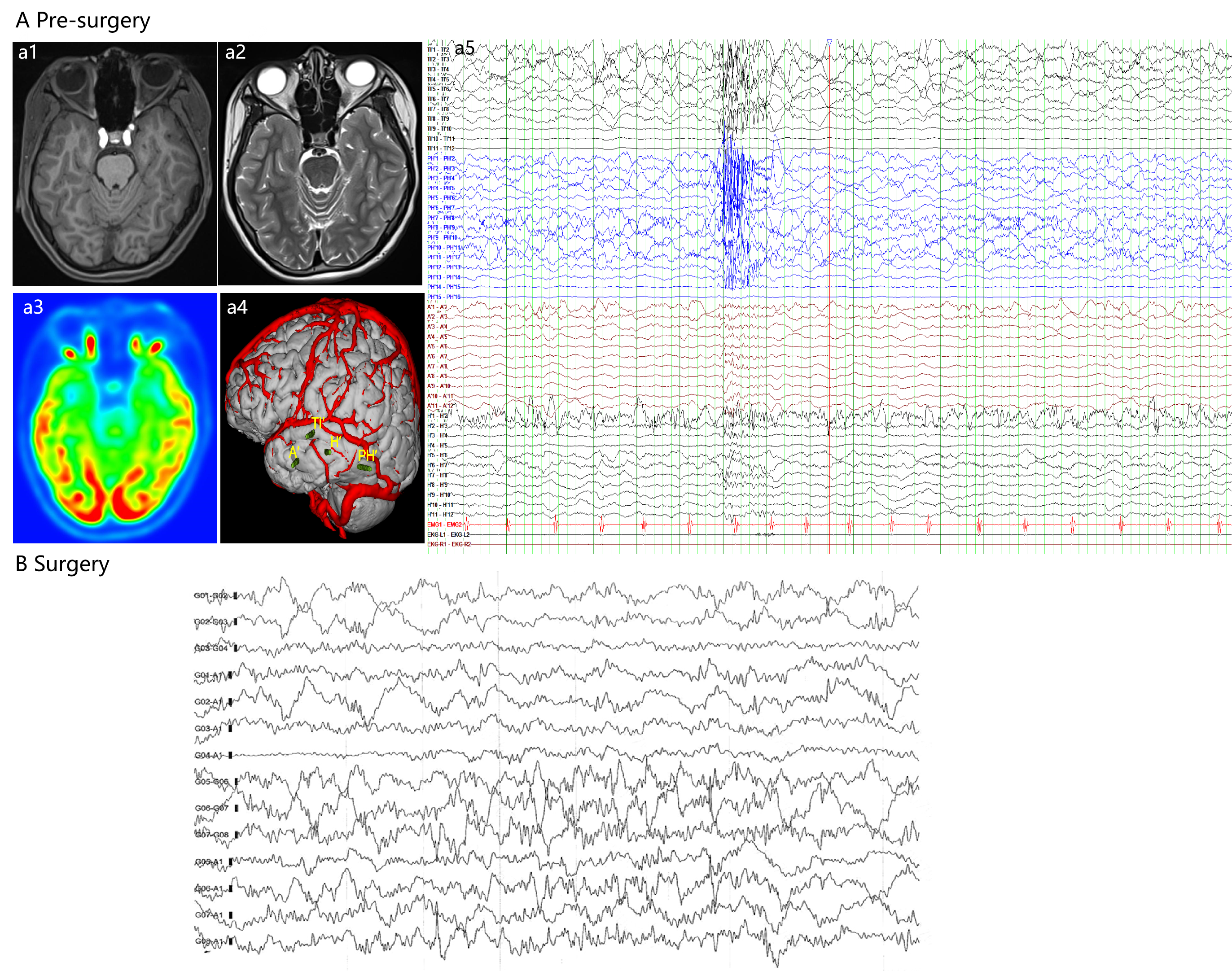


Fig. S1. Representative imaging and electrophysiological characteristics of the FCDI and Control groups. (A) Presurgical evaluation and confirmation of EZ and control. (a1-a2) The MRI findings are negative in patients with FCDI. (a3) PET-CT reveals hypometabolism in the left temporal lobe. (a4) Schematic diagram of electrode implantation in the left temporal lobe. (a5) The seizure-phase SEEG recorded an epileptic discharge in the left temporal lobe. (B) Temporal lobe epileptiform discharges recorded by intraoperative EcoG.

Fig. S2. Postoperative histopathological examination of the temporal neocortex.
Panels a4, a9, and a14 show NeuN immunohistochemical staining; all other panels show H&E staining. (a1,a6,a11) Partial area of the FCDIa temporal neocortex layers II-III neurons are arranged in a microcolumnar organization (arrows). (a2, a7,a12) Partial area of the FCDIc temporal neocortex layers II-III neurons are arranged in a microcolumnar organization (arrows) with a small number of partial regional neurons lost (not shown). (a3,a8,a13) FCDIb shows cortical disorganization in the temporal lobe neocortex, along with cortical thinning(arrow). (a4,a9,a14) NeuN staining demonstrates microcolumnar organization of neurons in layers II–III in FCD Ia (arrows).(a5,a10,a15) Normal temporal lobe neocortex. (a1-a5) Scale bars=500μm; (a6-a10) Scale bars=200μm;(a11-a15) Scale bars=100μm.


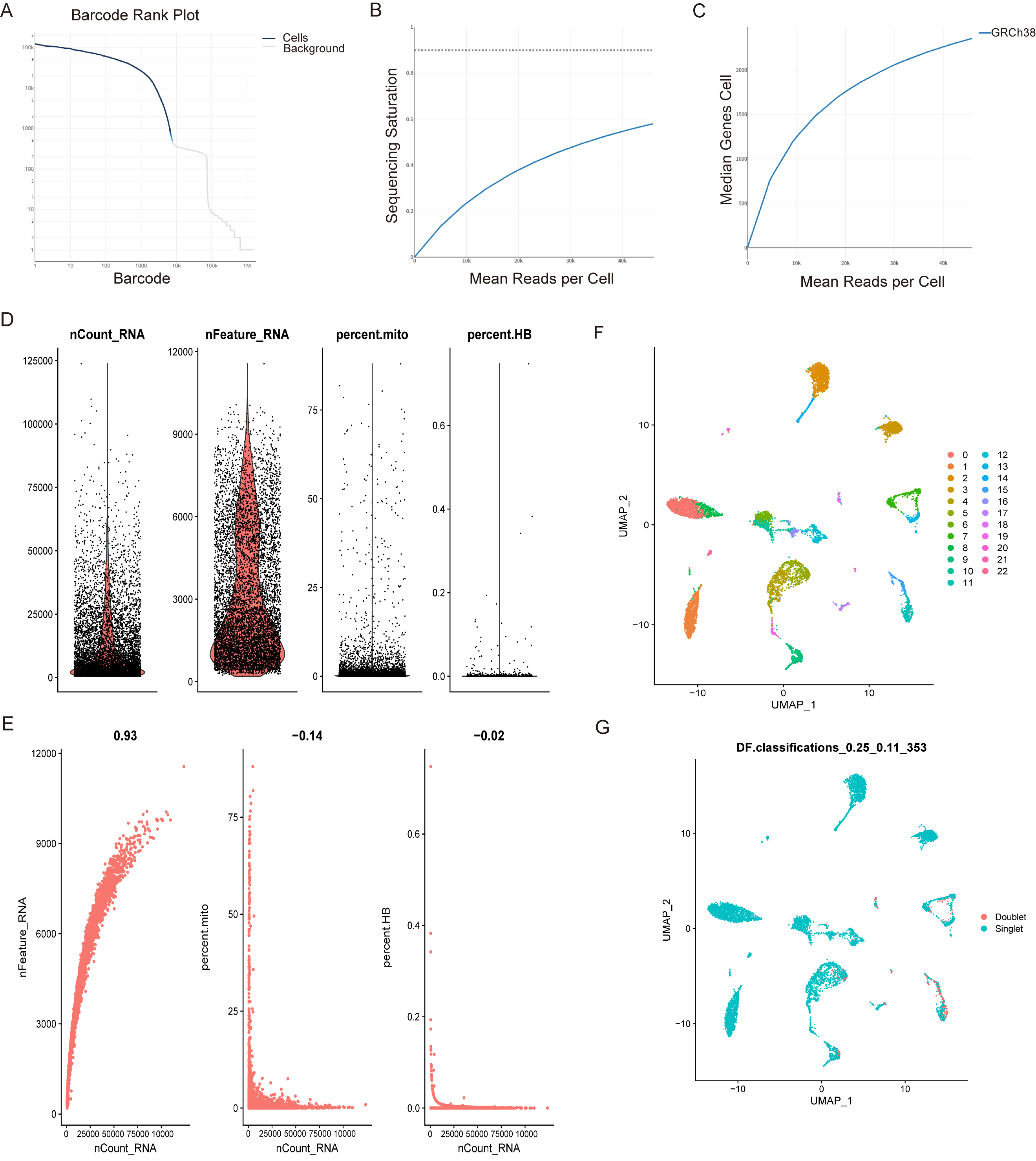


Fig. S3. snRNA-seq data quality control(A) Barcode rank plot. The blue curve indicates the number of cells detected and the grey curve is the background with clear demarcation. (B) Sequencing depth saturation plot. When the curve flattens near the endpoint, its slope can reflect the upper limit of the information gain achievable by further increasing the sequencing depth. (C) Saturation plot of median genes per cell. (D) Seurat visualizes the total number of RNA counts of the cells (nCount_RNA), the number of genes detected in each cell (nFeature_RNA), and the percentage of mitochondrial genes and red blood cell-related genes. (E) Seurat visualizes the total number of RNA counts in cells in relation to gene counts, mitochondrial genes, and red blood cell-related genes. (F) UMAP plot when doublet cells were not removed. (G) Visualization of doublet cells in UMAP plots.


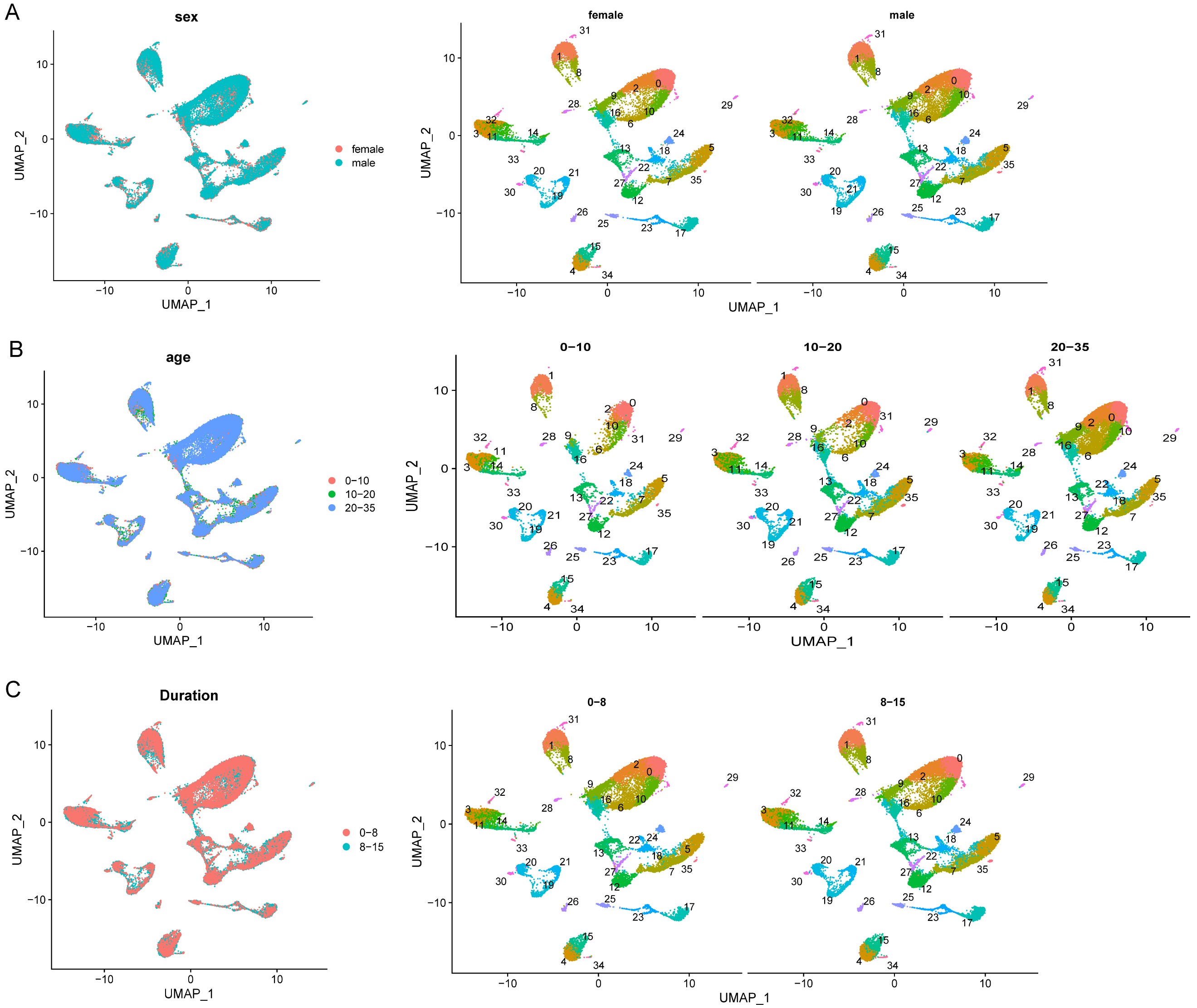


Fig.S4. Categorical variables of the snRNA-seq dataset. (A) UMAP visualization of the snRNA-seq dataset colored by sex. (B) UMAP visualization of the snRNA-seq dataset colored by age. (C) UMAP visualization of the snRNA-seq dataset colored by disease duration.


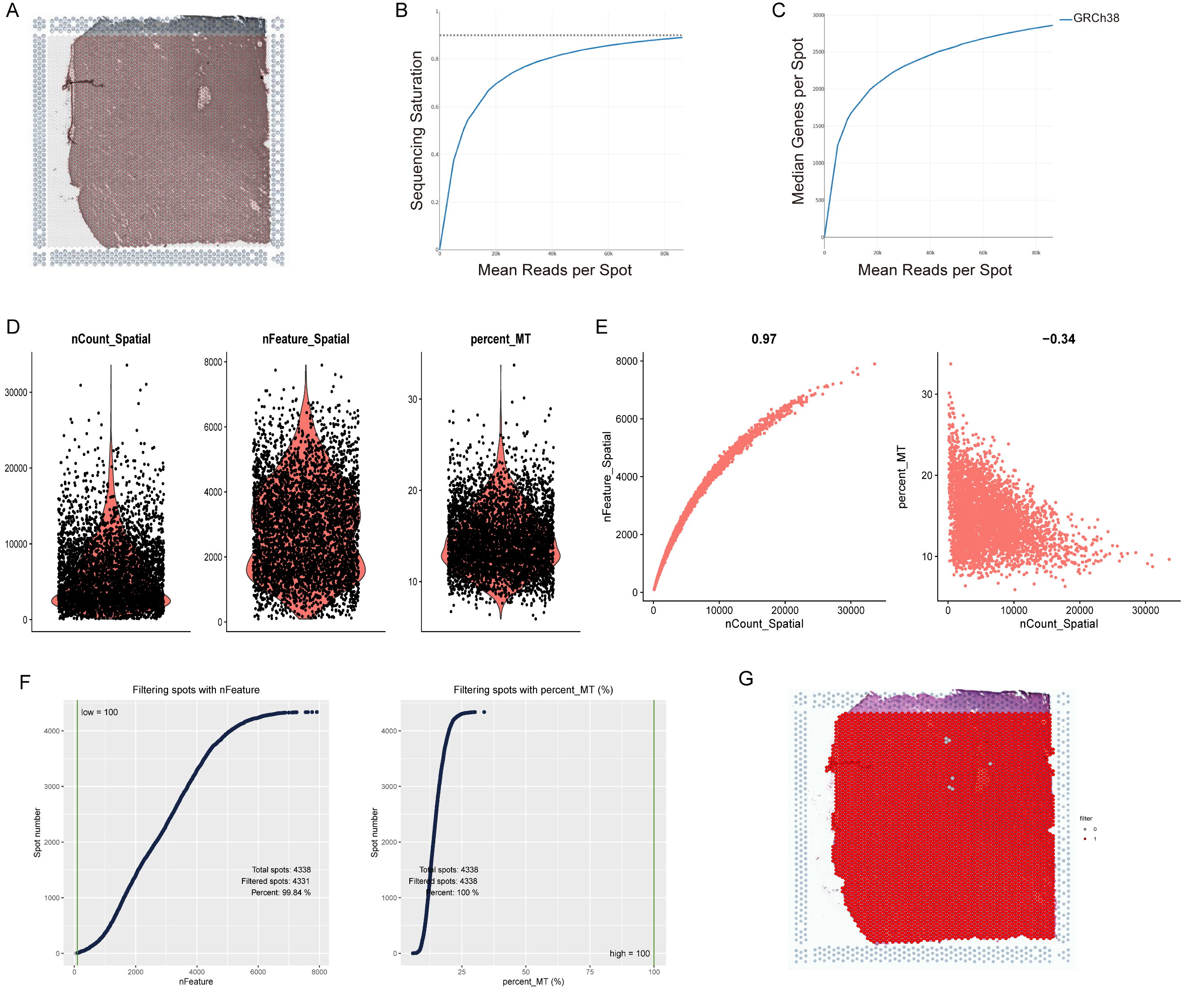


Fig.S5. ST-seq data quality control. (A) Tissue detection and fiducial alignment. (B) Sequencing depth saturation plot. (C) Saturation plot of median genes per spot. (D) Seurat visualizes the total RNA counts in the captured region (nCount_RNA), the number of genes detected in each spot (nFeature_RNA), and the proportion of mitochondrial genes. (E) Seurat visualizes the relationship between total RNA counts, the number of genes, and the proportion of mitochondrial genes. (F) Spot count after tissue filtering in Seurat. (G) Spatial distribution of spots after tissue filtering. Gray represents spots that were filtered out, and red represents the remaining spots after filtering.


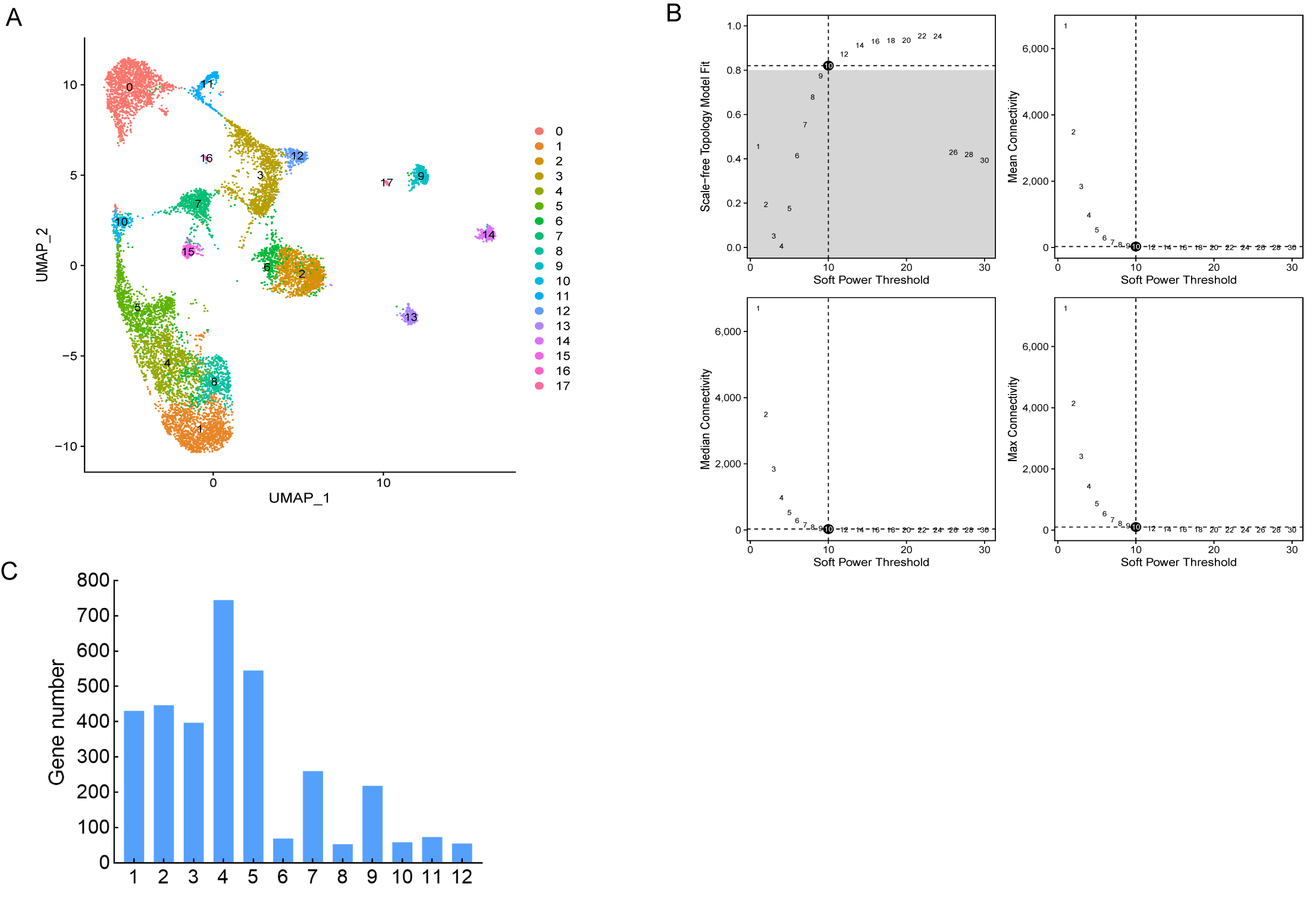


Fig.S6. hdWGCNA identification of gene modules in ENs. (A) UMAP plot of excitatory neurons after secondary dimensionality reduction clustering, with 18 clusters identified and nuclei colored by cluster. (B) Analysis of the scale-free index and mean connectivity for various soft-threshold powers (β). (C) The number of genes per module. (D) Distribution of co-expression module trait genes in UMAP.


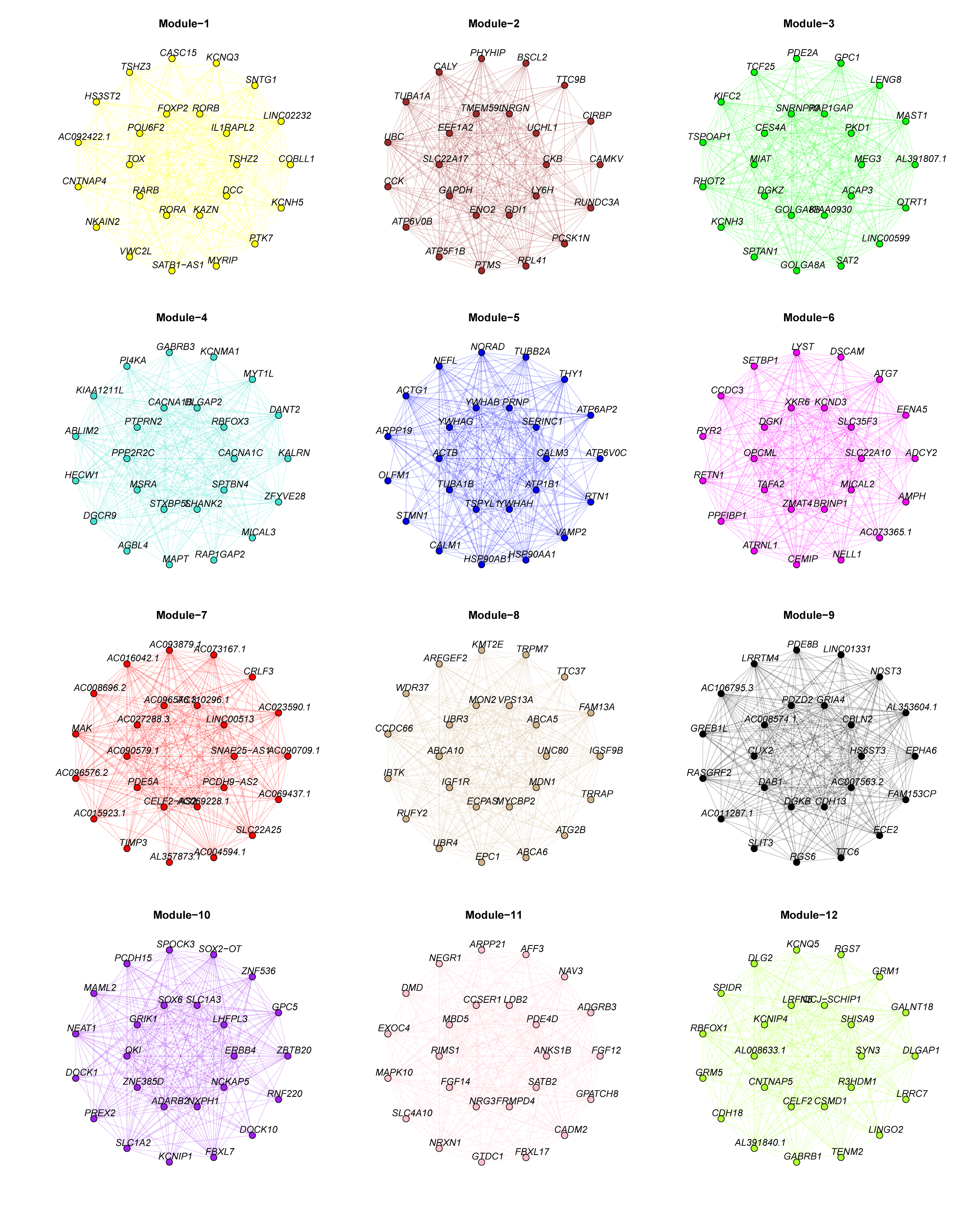


Fig.S7. hdWGCNA identifies co-expression modules and hub gene network in ENs


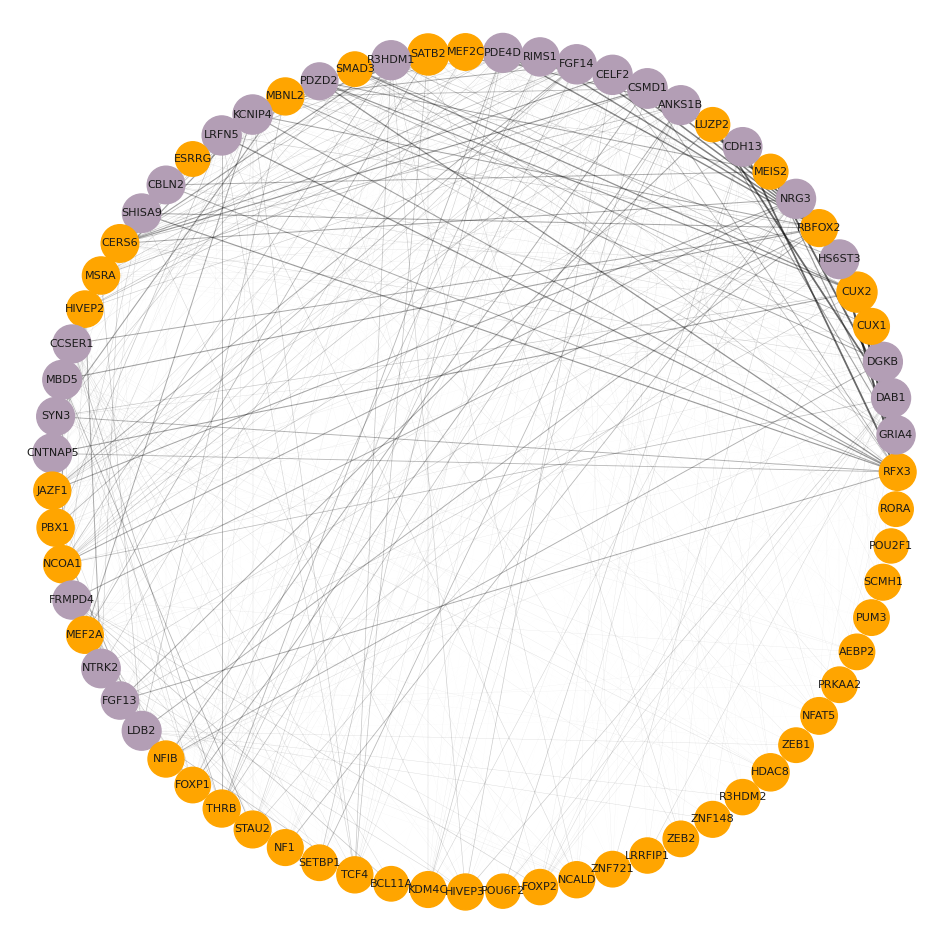


Fig.S8. TF-hub gene regulatory network. The genes within the yellow circle are TFs, the genes within the purple circle are core genes, and the black lines represent regulatory relationships.


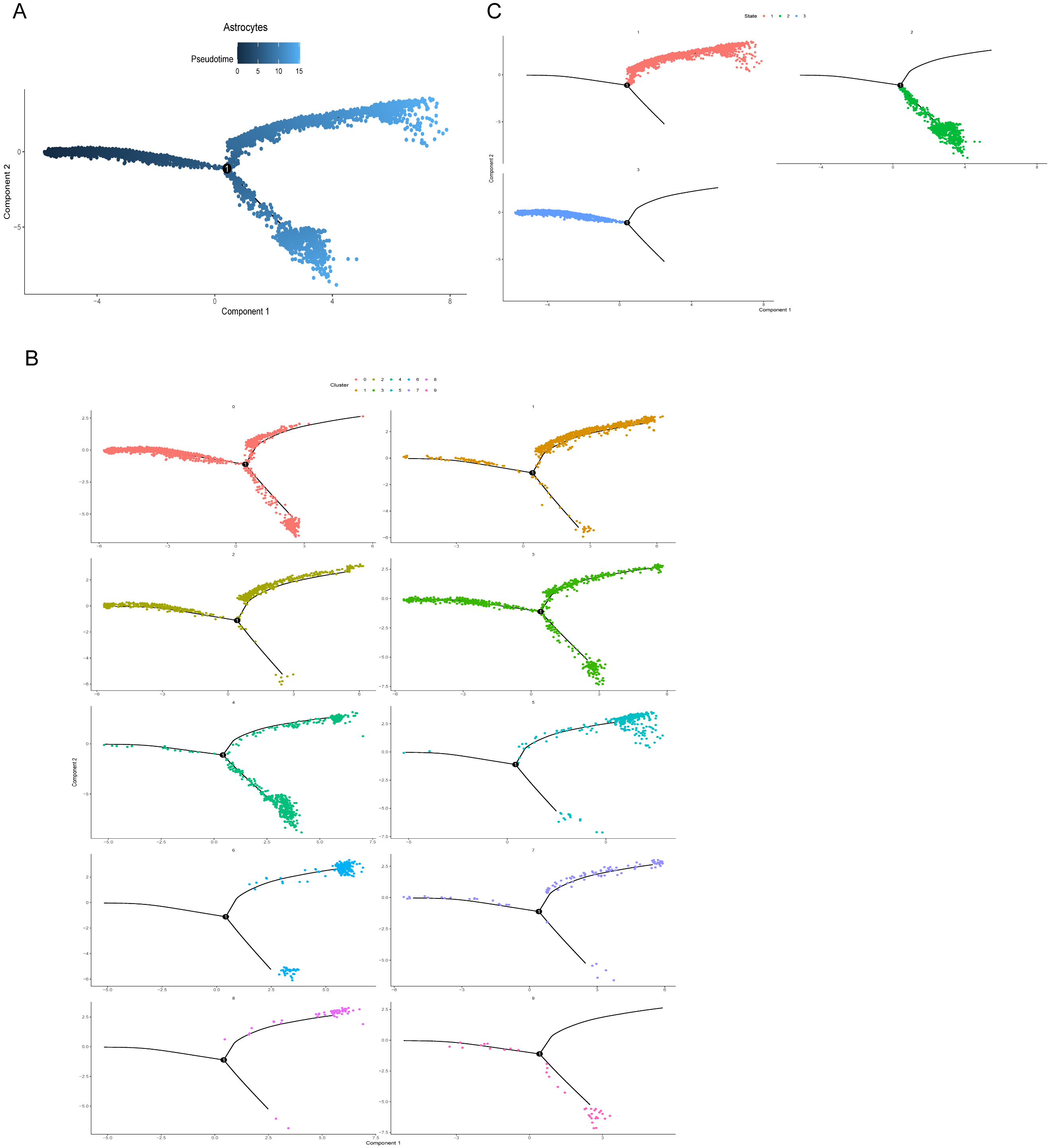


Fig.S9. Differentiation trajectory and state distribution of astrocyte subpopulations. (A) Pseudo-time trajectory of astrocyte cells. The color of each point in the figure corresponds to the pseudo-time value of the cell. Pseudo-time is commonly used in trajectory inference analysis to reflect the state of the cell during a temporal or developmental process. Cells with lower pseudo-time are shown in dark blue, while cells with higher pseudo-time are shown in light blue. The pseudo-time axis is typically used to infer different stages in the cell's development or transition process. (B)Facet plot of pseudo-time analysis results categorized by clusters. (C)Visualization of cell distribution in different states before and after branch points in pseudo-time analysis.


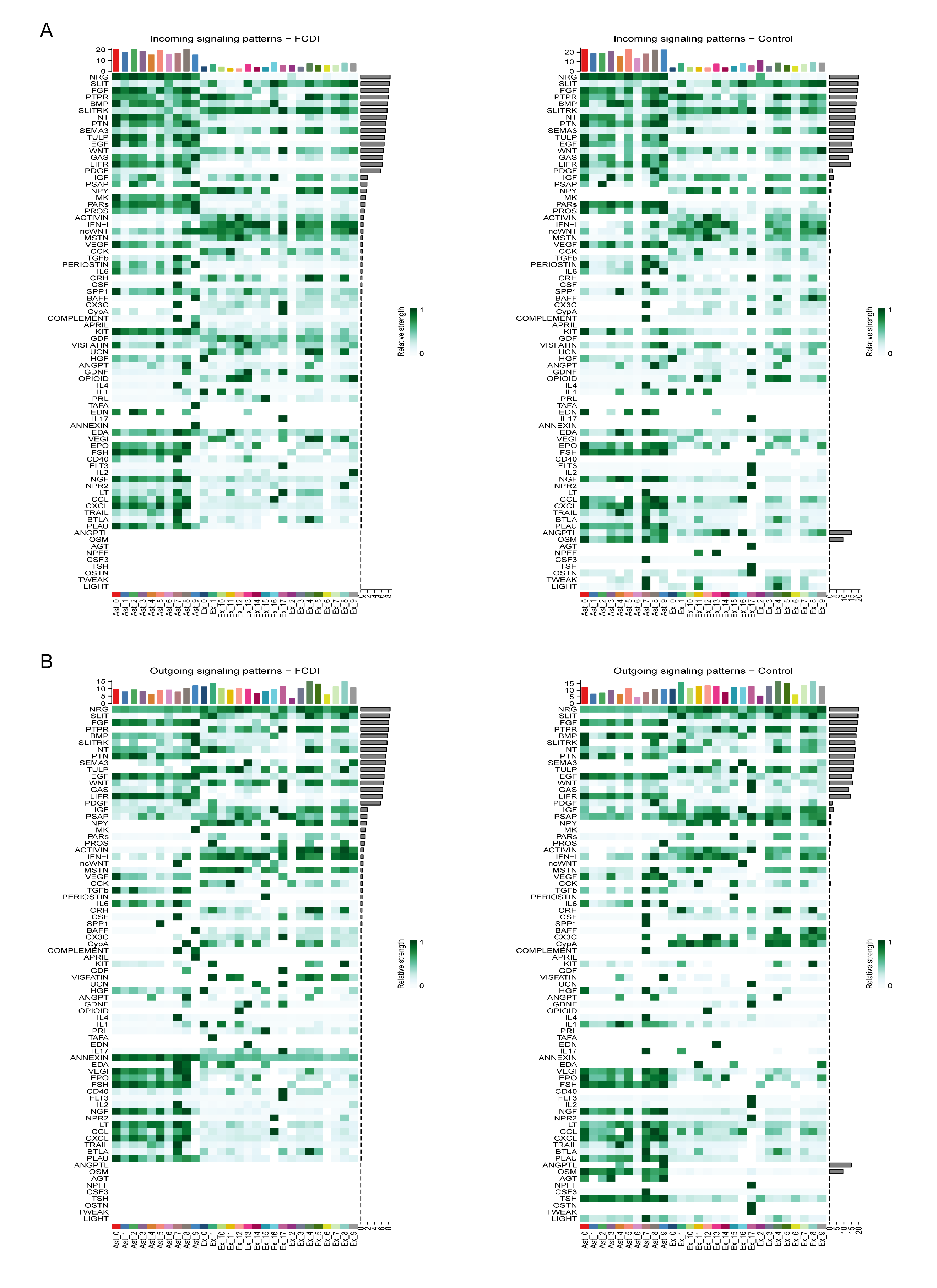


Fig.S10. Incoming and outgoing signals in the cell communication between ENs and Ast. (A) Heatmap of incoming signaling patterns. (B) Heatmap of outgoing signaling patterns. Rows and columns represent signaling pathways and cell subtypes, respectively. The color intensity indicates the relative strength of the signaling pathway among the given cells.
